# Synthesis and biological evaluation of the new 1,3-dimethylxanthine derivatives with thiazolidine-4-one scaffold

**DOI:** 10.1186/s13065-017-0241-0

**Published:** 2017-02-01

**Authors:** Sandra Constantin, Florentina Geanina Lupascu, Maria Apotrosoaei, Ioana Mirela Vasincu, Dan Lupascu, Frederic Buron, Sylvain Routier, Lenuta Profire

**Affiliations:** 1 The Department of Pharmaceutical Chemistry, The Faculty of Pharmacy, “Grigore T. Popa” University of Medicine and Pharmacy, No. 16 University Street, Iasi, 700115 Romania; 2 Institut de Chimie Organique et Analytique, ICOA, Univ Orleans, Orleans, France

**Keywords:** 1,3-Dimethylxanthine, 1,3-Thiazolidine-4-one, Spectral methods, Antioxidant effects

## Abstract

**Background:**

The xanthine structure has proved to be an important scaffold in the process of developing a wide variety of biologically active molecules such as bronchodilator, hypoglycemiant, anticancer and anti-inflammatory agents. It is known that hyperglycemia generates reactive oxygen species which are involved in the progression of diabetes mellitus and its complications. Therefore, the development of new compounds with antioxidant activity could be an important therapeutic strategy against this metabolic syndrome.

**Results:**

New thiazolidine-4-one derivatives with xanthine structure have been synthetized as potential antidiabetic drugs. The structure of the synthesized compounds was confirmed by using spectral methods (FT-IR, ^1^H-NMR, ^13^C-NMR, ^19^F-NMR, HRMS). Their antioxidant activity was evaluated using in vitro assays: DPPH and ABTS radical scavenging ability and phosphomolybdenum reducing antioxidant power assay. The developed compounds showed improved antioxidant effects in comparison to the parent compound, theophylline. In the case of both series, the intermediate (**5a–k**) and final compounds (**6a–k**), the aromatic substitution, especially in *para* position with halogens (fluoro, chloro), methyl and methoxy groups, was associated with an increase of the antioxidant effects.

**Conclusions:**

For several thiazolidine-4-one derivatives the antioxidant effect of was superior to that of their corresponding hydrazone derivatives. The most active compound was **6f** which registered the highest radical scavenging activity.

**Graphical abstract:**
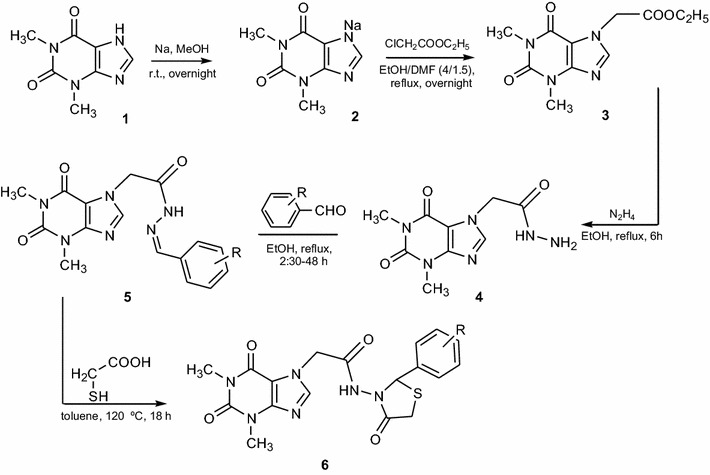
Design and synthesis of new thiazolidine-4-one derivatives.

**Electronic supplementary material:**

The online version of this article (doi:10.1186/s13065-017-0241-0) contains supplementary material, which is available to authorized users.

## Background

Discovered around 1888, xanthine scaffold, under the form of methylxanthine alkaloids, is naturally found in coffee (*Coffea arabica*) and tea (*Camellia sinensis*) and is associated with interesting biological activities, having bronchodilatatory (theophylline), diuretic (theobromine) and phychostimulant (caffeine) effects [1–3]. The new biologically active compounds such as bronchodilator [3], hypoglycemiant [4], anticancer [5] and anti-inflammatory [6] agents have been discovered by chemical modulation of this scaffold. An example of hypoglycemic agent is Linagliptin (Tradjenta^®^, Trajenta^®^), a DPP-4 inhibitor [7, 8] which has been used in the USA for the treatment of diabetes mellitus type 2 since 2011. Its additional antioxidant properties proved to be very useful in managing the vascular complications of diabetes (macrovascular-myocardial infarction, angina pectoris, stroke and microvascular-diabetic nephropathy and retinopathy, impotence, “diabetic foot”) [9].

Many recent research studies have focused on thiazolidine-4-one heterocycle, due to the role it plays in the synthesis [10] of new derivatives which showed significant biological activity as antidiabetic [11, 12], antioxidant [13–15], anticonvulsant [16], anticancer [17], anti-inflammatory, analgesic [18], antimicrobial, antifungal [19], antiviral [20], antihypertensive, antiarrhythmic [21], anti-mycobacterial [22] and antiparasitic [23] effect. Moreover, the thiazolidinediones developed from this scaffold are important drugs used in treatment of diabetes mellitus type 2. Three thiazolidinediones (pioglitazone, rosiglitazone and lobeglitazone) are approved for diabetes mellitus therapy [24]. Although these drugs are a very effective for reducing hyperglycemia, they are also associated with serious side effects such as hepatotoxicity, weight gain, macular edema and cardiovascular events [25, 26].

Diabetes mellitus is a chronic metabolic disorder considered a major health problem in the whole world. Every year 4 million people die from diabetes mellitus and 1.5 million new cases are diagnosed. This disease is characterized by hyperglycemia, a condition which, if not properly controlled, can lead to complications at the level of different organs. It mainly affects the eyes, the heart, the kidneys and the blood vessels. Hyperglycemia also generates reactive oxygen species (ROS) which can produce cell damages by means of different mechanism [27]. It has been proven that oxidative stress (an imbalance between the production of ROS and the scavenging ability of the body) holds a key role in the development of diabetes mellitus and its complications. The scavenging ability is closely related to the concentration of endogenous oxidative enzymes such as catalase, glutathione peroxidase and superoxide dismutase [28].

In order to develop new compounds with antioxidant effects and potential applications in antidiabetic therapy, new thiazolidine-4-one derivatives with xanthine structure have been synthesized. The structure of these compounds was proved by means of spectral methods (FT-IR, ^1^H-NMR, ^13^C-NMR, ^19^F-NMR, HRMS) and their antioxidant effects were evaluated using in vitro assays: DPPH and ABTS radical scavenging ability and phosphomolybdenum reducing antioxidant power assay.

## Results and discussion

### Chemistry

**Scheme 1:**
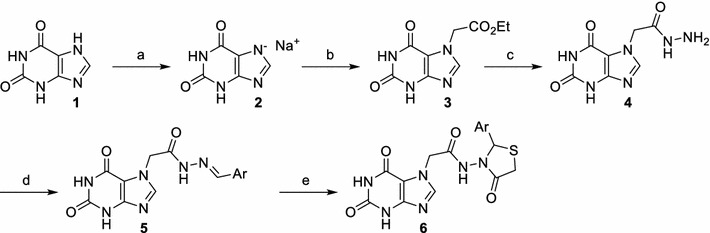
Synthesis of compounds **6a–j**. Reagents and conditions: (*a*) sodium, dry MeOH, r.t., overnight; (*b*) ethyl chloroacetate, EtOH/DMF (4:1.5), reflux, overnight; (*c*) hydrazine hydrate 64%, EtOH, reflux, 6 h; (*d*) aromatic aldehyde, EtOH, reflux, 2 h 30 min–48 h; (*e*) mercaptoacetic acid, toluene, heating 120 °C, 18 h

The new 1,3-thiazolidine-4-one derivatives were synthesized according to Scheme 1. Theophylline (1,3-dimethylxanthine) **1**, in the presence of sodium methoxide, gave the salt **2** in a quantitative yield; the salt in its turn reacted with ethyl chloroacetate and resulted in theophylline-ethyl acetate **3** [4]. The reaction of the compound **3** with an excess of hydrazine hydrate 64% resulted in a very good yield of theophylline hydrazide **4**. Then, the condensation of the compound **4** with different aromatic aldehydes led to the formation of the corresponding hydrazones **5a–k** in satisfying yields [29, 30]. Finally, the cyclization of hydrazones (**5a–k**) in the presence of mercaptoacetic acid had as result thiazolidine-4-one derivatives **6a–k** in moderate to excellent yields (Table 1). Totally were obtained 22 compounds from which 19 are new (8 hydrazones: **5b**, **5d**, **5e**, **5g–5k** and 11 thiazolidine-4-ones: **6a–k**).

**Table 1:** Synthesis of compounds **5** and **6**

Entry	No	Compound **5**	Yield (%)	Entry	No	Compound **6**	Yield (%)
1	**5a**		74	12	**6a**		50
2	**5b**		95	13	**6b**		30
3	**5c**		95	14	**6c**		29
4	**5d**		90	15	**6d**		28
5	**5e**		87	16	**6e**		33
6	**5f**		91	17	**6f**		37
7	**5g**		94	18	**6g**		50
8	**5h**		93	19	**6h**		50
9	**5i**		93	20	**6i**		6
10	**5j**		79	21	**6j**		54
11	**5k**		89	22	**6k**		52

The structure of the compounds was proved on the basis of the spectral data (IR, ^1^H-NMR,^13^C-NMR,^19^F-NMR, HRMS) provided in the “Experimental section” part of the paper. The IR and NMR spectral data for compounds **3** and **4** were previously presented [4].

The specific CH=N bond of hydrazone derivatives **5a–k** appeared in IR spectra in the region of 1544–1609 cm^−1^. Other specific bands appeared in the region of 1635–1671 cm^−1^ (CO–NH) and 3034–3110 cm^−1^ (–NH). The thiazolidine-4-one ring of the **6a–k** derivatives was identified in the IR spectra by specific bands of C=O and C–S bonds which appeared at 1682–1701 and 665–699 cm^−1^, respectively.

In the ^1^H-NMR spectra of hydrazones **5a–k** there were identified two sets of signals which corresponded to the two tautomer forms and were in dynamic equilibrium with each other. The proton from amide group (CO–NH) is responsible for the lactam-lactim tautomerism, obtaining two forms: the hydrazone (lactam form, HN–C=O) and the tautomer (lactim form, N=C–OH). The ratio between tautomers ranged between 9:1 and 7:3, depending on the compound. The proton of the azomethine group (N=CH) resonated as a singlet at 7.96–8.38 ppm for one form and at 8.06–8.55 ppm for the other form. The proton of the amide group (CO–NH) appeared as a singlet at 11.55–11.83 ppm in the case of the hydrazone and at 11.63–11.83 ppm in the case of the tautomer form. The tautomerism was proved by ^1^H-NMR at 80 °C, when one set of signals was recorded.

The success of the cyclization process which resulted in the formation of the thiazolidine-4-one ring, was proved by means of ^1^H-NMR data. The proton from the N–CH–S group was recorded as a singlet between 5.75 and 6.10 ppm while the protons of the methylene group (CH_2_–S) resonated as doublets of doublets or multiples in the interval between 3.63 and 3.80 ppm.

The structure of the synthesized compounds was strengthened by ^13^C-NMR data. The compounds **5a–k** had two azomethine groups (N=CH), one from the theophylline part and another one from the hydrazone chain. The carbon signals of these groups were observed between 140.3 and 148.8 ppm. Moreover, the carbons from the thiazolidine-4-one ring appeared at 56.9–61.7 ppm (N–CH–S) and 29.9–30.1 ppm (CH_2_–CO).

The fluorine atom from the structure of **5d** and **6d**, resonated in ^19^F-NMR spectra as a specific signal registered at −110.5 and −110.4 ppm in the case of the tautomer forms of hydrazone and at −110.2 ppm in the case of the thiazolidine-4-one derivatives.

The molecular mass of hydrazones **5a–k** and of the corresponding thiazolidine-4-one derivatives, **6a–k**, was probed by means of high resolution spectral mass. The spectral mass data coupled with the NMR data (^1^H-NMR, ^13^C-NMR, ^19^F-NMR) proved the proposed structure for all synthesized compounds.

### Biological evaluation

#### DPPH radical scavenging assay

2,2-Diphenyl-1-picrylhydrazyl (DPPH) assay is usually applied for the evaluation of the antioxidant activity of different compounds. The method is based on the reduction of DPPH, which is violet in methanol solution, to a pale yellow compound, under the action of an antioxidant (proton donating agent). The absorbance of the yellow form is measured at 517 nm [31, 32]. The DPPH radical scavenging ability (%) of the theophylline–acethydrazide derivatives **5a–k** was calculated at different concentrations (0.4, 0.8, 1.2, 1.6, 2.0 mg/mL). Higher values of the scavenging ability indicate a superior effectiveness of the scavenging radical potential. It was observed that the scavenging ability of the hydrazones increased with the concentration, the best inhibition rate being recorded at the highest concentration used (2 mg/mL). The most significant increase from 0.4 to 2 mg/mL was recorded for **5a** (R=H). At the highest concentration used (2.0 mg/mL) the inhibition rate ranged from 4.51 ± 0.36% for **5c** (R = 4-Cl) to 18.65 ± 0.43% for **5a** (R=H) (Table 2). The inhibition rate of **5a** was higher than that of theophylline (**1**, 12.14 ± 0.20%). The compounds **5d** (R=4-F), 11.65 ± 0.19% and **5g** (R=3-OCH_3_), 10.66 ± 0.19% showed a similar activity to theophylline. However, the hydrazone derivatives were less active than vitamin C which was used as positive control.

**Table 2:** The DPPH scavenging ability (%) of derivatives **5a–k** at 2 mg/mL

Compound	Scavenging ability (%)	Compound	Scavenging ability (%)
**5a**	18.65 ± 0.43	**5g**	10.66 ± 0.19
**5b**	9.79 ± 0.30	**5h**	4.01 ± 0.24
**5c**	4.51 ± 0.36	**5i**	9.08 ± 0.13
**5d**	11.65 ± 0.19	**5j**	5.90 ± 0.24
**5e**	5.90 ± 0.24	**5k**	5.56 ± 0.19
**5f**	5.64 ± 0.32		
**Theophylline**	12.14 ± 0.20	**Vitamin C** ^a^	81.62 ± 0.21

^a^0.04 mg/mL; Data are mean ± SD (n = 3, *p* < 0.05)

The scavenging ability of the theophylline-acethydrazide derivatives was improved by cyclization to the corresponding thiazolidine-4-one derivatives **6a–k**. Their scavenging ability at different concentrations (0.4, 0.8, 1.2, 1.6, 2.0 mg/mL) was higher than the value recorded for the corresponding hydrazones. The inhibition rate of **6a–k** was similar to the one of **5a–k** and increased with the concentration, the best inhibition rate being recorded at the highest concentration used (2 mg/mL). At this concentration the best inhibition rate was shown by **6c** (R=4-Cl) and **6k** (R=4-CH_3_), with vlues of 77.53 ± 0.47% (EC_50_ = 1.1640 ± 0.0123 mg/mL) and 68.28 ± 0.19% (EC_50_ = 1.4389 ± 0.0130 mg/mL), respectively. In comparison to theophylline (**1**) these compounds were about 6.5 times and six times more active. The most promising compound seemed to be **6f** (R=4-OCH_3_), which had an inhibition rate of 64.50 ± 0.59% at 0.3 mg/mL, showing the best EC_50_ value (0.2212 ± 0.0011 mg/mL) (Table 3). These data supported the conclusion that the presence of the methoxy, chloro and methyl group in the *para* position of the phenyl ring of the thiazolidine-4-one scaffold had a good influence on the radical scavenging activity.

**Table 3:** The DPPH scavenging ability (%) at 2 mg/mL and EC_50_ (mg/mL) of **6a–k**

Compound	Scavenging ability (%)	Compound	Scavenging ability (%)
**6a**	27.99 ± 0.49	**6g**	35.05 ± 0.32
**6b**	13.11 ± 0.12	**6h**	24.60 ± 0.31
**6c**	77.53 ± 0.47^a^	**6i**	28.32 ± 0.18
**6d**	33.47 ± 0.42	**6j**	38.39 ± 0.28
**6e**	37.49 ± 0.45	**6k**	68.28 ± 0.19^c^
**6f** ^e^	64.50 ± 0.59^b^		
**Theophylline**	12.14 ± 0.20	**Vitamin C** ^f^	81.62 ± 0.21^d^

Data are mean ± SD (n = 3, *p* < 0.05)

EC_50_ (mg/mL): ^a^ 1.1640 ± 0.0123, ^b^ 0.2212 ± 0.0011, ^c^ 1.4389 ± 0.0130, ^d^ 0.0083 ± 0.0002, ^e^ 0.3 mg/mL; ^f^ 0.04 mg/mL

A good influence was also showed by the presence of the fluoro group in *para* position and of the methoxy group in *ortho* and *meta* position; the corresponding compounds **6d** (R=4-F), **6e** (R=2-OCH_3_), **6g** (R=3-OCH_3_) and **6j** (2,3-OCH_3_) being about three time more active than theophylline. However all tested compounds were less active than Vitamin C used as positive control.

#### ABTS radical scavenging ability

The radical of ABTS (2,2′-azino-bis-(3-ethylbenzothiazoline-6-sulfonic acid)), a blue chromophore (ABTS^·+^), was generated by oxidation with potassium persulfate. In the presence of hydrogen donating compounds, ABTS^·+^ was reduced and the corresponding form was quantitative measured by recording the absorbance value at 734 nm [33]. The scavenging ability of **5a–k** and **6a–k** at different concentrations (0.25, 0.5, 0.75, 1.0 mg/mL) was presented in Tables 4 and 6. In the theophylline-acethydrazide series the most active compounds were **5d** (R=4-F) and **5b** (R=4-Br), registering EC_50_ values of 0.2084 ± 0.0013 and 0.3662 ± 0.0030 mg/mL, respectively (Table 5). A good activity was also shown by **5c** (R=4-Cl), **5i** (R=2,4-OCH_3_) and **5k** (R=4-CH_3_).

**Table 4:** The ABTS scavenging ability (%) of derivatives **5a–k**

Compound	Scavenging ability (%)	Compound	Scavenging ability (%)
**5a** ^a^	20.65 ± 0.26	**5g** ^a^	23.83 ± 0.43
**5b** ^b^	70.20 ± 0.11	**5h** ^a^	22.09 ± 0.23
**5c** ^a^	62.74 ± 0.48	**5i** ^a^	66.30 ± 0.32
**5d** ^c^	57.39 ± 0.32	**5j** ^a^	26.30 ± 0.31
**5e** ^a^	27.21 ± 0.12	**5k** ^a^	76.16 ± 0.45
**5f** ^a^	29.21 ± 0.27		
**Theophylline** ^a^	25.97 ± 0.27	**Vitamin C** ^d^	78.42 ± 0.40

^a^1 mg/mL; ^b^ 0.5 mg/mL; ^c^ 0.25 mg/mL; ^d^ 0.004 mg/mL; Data are mean ± SD (n = 3, *p* < 0.05)

**Table 5:** The ABTS scavenging ability (EC_50_, mg/mL) of the most active compounds

Compound	EC_50_ (mg/mL)	Compound	EC_50_ (mg/mL)
**5b**	0.3362 ± 0.0030	**5i**	0.6718 ± 0.0026
**5c**	0.7187 ± 0.0039	**5k**	0.4224 ± 0.0040
**5d**	0.2084 ± 0.0013	**Vitamin C**	0.0028 ± 0.0001

Data are mean ± SD (n = 3, *p* < 0.05)

At a concentration of 1 mg/mL the most active compounds were **6d** (R=4-F) and **6f** (R=4-OCH_3_) for which the scavenging ability was 90.05 ± 0.07 and 73.43 ± 0.56% (Table 6). A good scavenging ability was also shown by **6e** (R=2-OCH_3_) and **6k** (R=4-CH_3_). The data supported the conclusion that fluoro, methoxy (*ortho*, *meta*) and the methyl group exercised a positive influence on the ABTS scavenging activity. The EC_50_ values for these compounds were presented in Table 7. All compounds were less active than positive control.

**Table 6:** The ABTS scavenging ability (%) of derivatives **6a–k**

Sample	Scavenging ability (%)	Sample	Scavenging ability (%)
**6a**	30.68 ± 0.09	**6g**	29.39 ± 0.15
**6b**	12.95 ± 0.28	**6h**	26.43 ± 0.15
**6c**	12.59 ± 0.31	**6i**	32.03 ± 0.14
**6d**	90.05 ± 0.07	**6j**	47.13 ± 0.12
**6e**	66.00 ± 0.12	**6k**	57.36 ± 0.11
**6f**	73.43 ± 0.56		
**Theophylline**	25.97 ± 0.27	**Vitamin C** ^a^	78.42 ± 0.40

^a^0.004 mg/mL; Data are mean ± SD (n = 3, *p* < 0.05)

**Table 7:** The ABTS scavenging ability (EC_50_, mg/mL) of the most active compounds

Compound	EC_50_ (mg/mL)	Compound	EC_50_ (mg/mL)
**6d**	0.8352 ± 0.0005	**6f**	0.3805 ± 0.0032
**6e**	0.5880 ± 0.0017	**6k**	0.6789 ± 0.0024
**Vitamin C**	0.0028 ± 0.0001		

Data are mean ± SD (n = 3, *p* < 0.05)

For some thiazolidin-4-one derivatives (**6a**, **6e**, **6f**, **6g**, **6h**, **6j**) the ABTS scavenging activity was improved in comparison to that of the corresponding hydrazone derivatives at concentration of 1 mg/mL.

#### Phosphomolybdenum reducing antioxidant power (PRAP) assay

Phosphomolybdenum reducing antioxidant assay, known as total antioxidant capacity assay, is a spectrophotometric method based on the formation of green colored phosphomolybdenum complex after the reduction of Mo(VI) to Mo(V) under the action of electron donating compounds in acidic medium [34]. An increase in optical density means a better total antioxidant capacity.

**Fig. 1:**
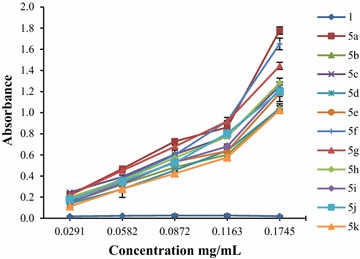
The absorbance of derivatives **5a–k**

**Fig. 2:**
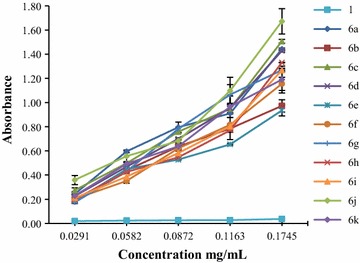
The absorbance of derivatives **6a–k**

In Figs. 1 and 2 there were presented the absorbance values of the tested compounds (**5a–k**, **6a–k**) at different concentrations (0.0291, 0.0582, 0.0872, 0.1163 and 0.1745 mg/mL). As we expected, the absorbance of the tested compounds increased with the concentration, the highest value of absorbance/activity being recorded at 0.1745 mg/mL, the highest concentration used. The data expressed as EC_50_ values (mg/mL) were shown in Tables 8 and 9.

**Table 8:** The phosphomolybdenum reducing antioxidant power (EC_50_, mg/mL) of **5a–k**

Compound	EC_50_ (mg/mL)	Compound	EC_50_ (mg/mL)
**5a**	0.0618 ± 0.0007	**5g**	0.0645 ± 0.0009
**5b**	0.0912 ± 0.0016	**5h**	0.0773 ± 0.0003
**5c**	0.0731 ± 0.0016	**5i**	0.0826 ± 0.0020
**5d**	0.0944 ± 0.0018	**5j**	0.0831 ± 0.0007
**5e**	0.0824 ± 0.0012	**5k**	0.1015 ± 0.0045
**5f**	0.0746 ± 0.0003		
**Theophylline**	nd	**Vitamin C**	0.0148 ± 0.0001

Data are mean ± SD (n = 3, *p* < 0.05)

**Table 9:** The phosphomolybdenum reducing antioxidant power (EC_50_, mg/mL) of **6a–k**

Compound	EC_50_ (mg/mL)	Compound	EC_50_ (mg/mL)
**6a**	0.0503 ± 0.0008	**6g**	0.0640 ± 0.0003
**6b**	0.0668 ± 0.0017	**6h**	0.0760 ± 0.0015
**6c**	0.0585 ± 0.0007	**6i**	0.0735 ± 0.0016
**6d**	0.0620 ± 0.0023	**6j**	0.0509 ± 0.0037
**6e**	0.0764 ± 0.0042	**6k**	0.0597 ± 0.0018
**6f**	0.0742 ± 0.0007		
**Theophylline**	nd	**Vitamin C**	0.0148 ± 0.0001

Data are mean ± SD (n = 3, *p* < 0.05)

*nd* undetected

In the theophylline-acethydrazide serie (**5a–k**) all tested compounds were more active than theophylline (**1**). The most active were **5a** (R=H, EC_50_ = 0.0618 ± 0.0007) and **5g** (R=3-OCH_3_, EC_50_ = 0.0645 ± 0.0009) (Table 8).

Some of thiazolidine-4-one derivatives showed an antioxidant capacity higher than that of the hydrazone derivatives, which proved that the presence of thiazolidine ring had a significant effect on the phosphomolybdenum reducing antioxidant power (Fig. 2). The most active compounds were **6a** (EC_50_ = 0.0503 ± 0.0008), **6j** (EC_50_ = 0.0509 ± 0.0037), **6c** (EC_50_ = 0.0585 ± 0.0007) and **6k** (EC_50_ = 0.0597 ± 0.0018). These data supported that the best substitution of the aromatic ring attached to a thiazolidine-4-one cycle was represented by hydrogen (**6a**), 3,5-dimethoxy (**6j**), 4-chloro (**6c**) and 4-methyl (**6k**), respectively (Table 9). In comparison to vitamin C (EC_50_ = 0.0148 ± 0.0001), used as positive control, these compounds were 3.4–4 times less active.

### Experimental section

#### General procedures

The melting points were measured by using a Buchi Melting Point B-540 apparatus and they were uncorrected. The FT-IR spectra were recorded on a Thermo-Nicolet AVATAR 320 AEK0200713 FT-IR Spectrometer, at a resolution of 4 cm^−1^ after six scans in the 4000–500 cm^−1^. The spectra processing was carried out with the Omnic Spectra Software. The ^1^H-NMR (250, 400 MHz), ^13^C-NMR (63, 101 MHz) and ^19^F-NMR (376 MHz) spectra were obtained on two types of Bruker Avance spectrometer: 250 and 400 MHz, using tetramethylsilane as internal standard and DMSO-d_6_ and CDCl_3_ as solvents. The chemical shifts were shown in δ values (ppm). The mass spectra were registered by using a BrukerMaXis Ultra-High Resolution Quadrupole Time-of-Flight Mass Spectrometer. The reactions were monitored by TLC, using pre-coated Kieselgel 60 F254 plates (Merck, Whitehouse Station, NJ, USA) and the compounds were visualized using UV light. The absorbance for biological assays was measured using a GBC Cintra 2010 UV–VIS spectrophotometer at different wavelengths: 517, 734 and 695 nm. The values were recorded in Cintral Software.

#### Synthetic procedures

##### The synthesis of hydrazide derivatives (**5a–k**)

The general procedure used for the synthesis of theophylline-acethydrazide derivatives and a part of their physical and chemical characteristics were described in our previous papers [29, 30]. The synthesis of the theophylline sodium salt **2**, theophylline ethyl acetate **3** and theophylline acetyl-hydrazine **4**, used as intermediaries in the synthesis of hydrazone derivatives **5a–k** was performed according to the literature procedure and some of their physical and chemical characteristics were presented in our previous paper [4].

*N*-*Benzylidene*-*2*-*(1,3*-*dimethylxanthin*-*7*-*yl)acethydrazide* (**5a**) Tautomeric mixture (8:2). ^13^C-NMR (101 MHz, DMSO-*d6*): δ = 168.3/163.3(C_q_), 154.9 (C_q_), 151.4 (C_q_), 148.3/148.4 (C_q_), 144.7/147.8 (CH=N), 144.1/144.2 (CH=N), 134.3/134.4 (C_q_), 130.5/130.6 (CH_Ar_), 129.3/129.2 (CH_Ar_), 127.3/127.5 (CH_Ar_), 107.1/106.8 (C_q_), 47.8/47.9 (N–CH_2_–CO), 29.9 (N–CH_3_), 27.8/27.9 (N–CH_3_); HRMS (EI-MS): *m/z* calculated for C_16_H_17_N_6_O_3_ [M + H]^+^ 341.13567, found 341.13566.

*N*-*(4*-*Bromobenzylidene)*-*2*-*(1,3*-*dimethylxanthin*-*7*-*yl)acethydrazide* (**5b**) Tautomeric mixture (8:2). ^13^C-NMR (101 MHz, DMSO-*d6*): δ = 168.5 (2 × C_q_), 154.9 (C_q_), 151.4 (C_q_), 148.2/148.4 (C_q_), 144.0/144.1 (CH=N), 143.5/146.6 (CH=N), 133.5/133.7 (C_q_), 132.2 (CH_Ar_), 129.2/129.4 (CH_Ar_), 123.8/123.9 (C_q_), 107.1/106.8 (C_q_), 47.8/47.9 (N–CH_2_–CO), 29.8 (N–CH_3_), 27.8 (N–CH_3_); HRMS (EI-MS): *m/z* calculated for C_16_H_16_BrN_6_O_3_ [M + H]^+^ 419.04618, found 419.04610.

*N*-*(4*-*Chlorobenzylidene)*-*2*-*(1,3*-*dimethylxanthin*-*7*-*yl)acethydrazide* (**5c**) Tautomeric mixture (8:2). ^13^C-NMR (101 MHz, DMSO-*d6*): δ = 168.5/163.4 (C_q_), 154.9 (C_q_), 151.5 (C_q_), 148.3/148.4 (C_q_), 144.1/144.2 (CH=N), 143.5/146.5 (CH=N), 135.0/135.1 (C_q_), 133.3/133.4 (C_q_), 129.4 (CH_Ar_), 129.0/129.2 (CH_Ar_), 107.2/106.9 (C_q_), 47.8/48.0 (N–CH_2_–CO), 29.9 (N–CH_3_), 27.9/27.8 (N–CH_3_); HRMS (EI-MS): *m/z* calculated for C_16_H_16_ClN_6_O_3_ [M + H]^+^ 375.09669, found 375.09641.

*N*-*(4*-*Fluorobenzylidene)*-*2*-*(1,3*-*dimethylxanthin*-*7*-*yl)acethydrazide* (**5d**) Tautomeric mixture (8:2). Yield 90%, white solid, m.p. 284–285 °C; IR (ATR diamond, cm^−1^): 3110 (–NH–), 2971 (CH_Ar_), 1636 (–CO–NH–), 1600 (–CH=N), 1226 (C–F); ^1^H-RMN (400 MHz, DMSO-*d6*): δ = 11.78 (s, 1H, CO–NH), 8.05 (d, J = 2.7 Hz, 2H, CH=N)/8.22, 8.07 (s, 2H, CH=N), 7.83–7.73 (m, 2H, Ar–H), 7.30 (t, *J* = 8.3 Hz, 2H, Ar–H), 5.55/5.12 (s, 2H, CH_2_–CO), 3.45 (s, 3H, N–CH_3_), 3.20 (s, 3H, N–CH_3_); ^13^C-NMR (101 MHz, CDCl_3_): δ = 173.1/169.4 (C_q_), 159.6/168.0 (C_q_), 156.2/167.0 (C_q_), 153.0/153.1 (C_q_), 148.8 (CH=N), 148.4 (CH=N), 135.7/135.8 (C_q_), 135.6/135.8 (C_q_), 134.3/134.5 (CH_Ar_), 134.3/134.5 (CH_Ar_), 121.2 (CH_Ar_), 121.0 (CH_Ar_), 111.9/111.6 (C_q_), 52.5/52.6 (N–CH_2_–CO), 34.6 (N–CH_3_), 32.6/32.7 (N–CH_3_); ^19^F-RMN (376 MHz, CDCl_3_): δ = −110.5/−110.4 (s, 1F, Ar–F), HRMS (EI-MS): *m/z* calculated for C_16_H_16_FN_6_O_3_ [M + H]^+^ 359.12624, found 359.12651.

*N*-*(2*-*Methoxybenzylidene)*-*2*-*(1,3*-*dimethylxanthin*-*7*-*yl)acethydrazide* (**5e**) Tautomeric mixture (8:2). Yield 86%, white solid, m.p. 274–275 °C; IR (ATR diamond, cm^−1^): 3034 (–NH–), 2974 (CH_Ar_), 1648 (–CO–NH–), 1600 (–CH=N), 1114 (O–C); ^1^H-RMN (400 MHz, DMSO-*d6*): δ = 11.70 (s, 1H, CO–NH), 8.38/8.55 (s, 1H, CH=N), 8.04/8.06 (s, 1H, CH=N), 7.86 (d, *J* = 7.6 Hz, 1H, Ar–H)/7.76 (d, *J* = 7.5 Hz, 1H, Ar–H), 7.40 (t, *J* = 7.2 Hz, 1H, Ar–H), 7.09 (d, *J* = 8.3 Hz, 1H, Ar–H), 7.00 (t, *J* = 7.6 Hz, 1H, Ar–H), 5.52/5.09 (s, 2H, CH_2_–CO), 3.85 (s, 3H, O–CH_3_), 3.43 (s, 3H, N–CH_3_), 3.18 (s, 3H, N–CH_3_); ^13^C-NMR (101 MHz, DMSO): δ = 168.2/163.1 (C_q_), 158.1/158.2 (C_q_), 154.8 (C_q_), 151.4 (C_q_), 148.2/148.4 (C_q_), 144.0/144.1 (CH=N), 140.3/143.3 (CH=N), 132.0/132.1 (CH_Ar_), 125.8/125.9 (CH_Ar_), 122.2/122.3 (C_q_), 121.1 (CH_Ar_), 112.2 (CH_Ar_), 107.1/106.8 (C_q_), 56.1 (O–CH_3_), 47.8/47.9 (N–CH_2_–CO), 29.8 (N–CH_3_), 27.8 (N–CH_3_); HRMS (EI-MS): *m/z* calculated for C_17_H_19_N_6_O_4_ [M + H]^+^ 371.14623, found 371.14591.

*N*-*(4*-*Methoxybenzylidene)*-*2*-*(1,3*-*dimethylxanthin*-*7*-*yl)acethydrazide* (**5f**) Tautomeric mixture (8:2). Yield 91%, white solid, m.p. 250–251 °C; IR (ATR diamond, cm^−1^): 3094 (–NH–), 2961 (CH_Ar_), 1659 (–CO–NH–), 1606 (–CH=N), 1125 (O–C); ^1^H-RMN (400 MHz, DMSO-*d6*): δ = 11.63 (s, 1H, CO–NH), 8.06/8.16 (s, 1H, CH=N), 7.99/8.07 (s, 1H, CH=N), 7.67 (d, *J* = 8.7 Hz, 2H, Ar–H)/7.64 (d, *J* = 9.0 Hz, 2H, Ar–H), 7.02 (d, *J* = 8.7 Hz, 2H, Ar–H), 5.53/5.10 (s, 2H, CH_2_–CO), 3.80 (d, *J* = 3.3 Hz, 3H, O–CH_3_), 3.46 (s, 3H, N–CH_3_), 3.20 (s, 3H, N–CH_3_); ^13^C-NMR (101 MHz, DMSO-*d6*): δ = 168.1/163.0 (C_q_), 161.2/161.3 (C_q_), 154.9 (C_q_), 151.4 (C_q_), 148.3/147.6 (C_q_), 144.6 (CH=N), 144.1/144.2 (CH=N), 128.9/129.2 (CH_Ar_), 126.2/126.9 (C_q_), 114.8 (CH_Ar_), 107.1/106.8 (C_q_), 55.7 (O–CH_3_), 47.8/47.9 (N–CH_2_–CO), 29.9 (N–CH_3_), 27.8/27.9 (N–CH_3_); HRMS (EI-MS): *m/z* calculated for C_17_H_19_N_6_O_4_ [M + H]^+^ 371.14623, found 371.14586.

*N*-*(3*-*Methoxybenzylidene)*-*2*-*(1,3*-*dimethylxanthin*-*7*-*yl)acethydrazide* (**5** **g**) Tautomeric mixture (8:2). Yield 94%, white solid, m.p. 238–239 °C; IR (ATR diamond, cm^−1^): 3075 (–NH–), 2965 (CH_Ar_), 1660 (–CO–NH–), 1598 (–CH=N), 1157 (C–O); ^1^H-RMN (400 MHz, DMSO-*d6*): δ = 11.77 (s, 1H, CO–NH), 8.06/8.19 (s, 1H, CH=N), 8.02/8.07 (s, 1H, CH=N), 7.40–7.33 (m, 1H, Ar–H), 7.28 (t, *J* = 10.0 Hz, 2H, Ar–H), 7.01 (d, *J* = 7.7 Hz, 1H, Ar–H), 5.55/5.12 (s, 2H, CH_2_–CO), 3.80/3.79 (s, 3H, O–CH_3_), 3.46 (s, 3H, N–CH_3_), 3.20 (s, 3H, N–CH_3_); ^13^C-NMR (101 MHz, DMSO-*d6*): δ = 168.4/163.3 (C_q_), 160.0/159.9 (C_q_), 154.9 (C_q_), 151.5 (C_q_), 148.3/148.4 (C_q_), 144.6/147.6 (CH=N), 144.1/144.2 (CH=N), 135.7/135.8 (C_q_), 130.4 (CH_Ar_), 120.0/120.4 (CH_Ar_), 116.5/116.74 (CH_Ar_), 111.9 (CH_Ar_), 107.1/106.8 (C_q_), 55.6 (O–CH_3_), 47.8/47.9 (N–CH_2_–CO), 29.9 (N–CH_3_), 27.8 (N–CH_3_); HRMS (EI-MS): *m/z* calculated for C_17_H_19_N_6_O_4_ [M + H]^+^ 371.14623, found 371.14612.

*N*-*(2,3*-*Dimethoxybenzylidene)*-*2*-*(1,3*-*dimethylxanthin*-*7*-*yl)acethydrazide* (**5** **h**) Tautomeric mixture (8:2). Yield 93%, white solid, m.p. 252–253 °C; IR (ATR diamond, cm^−1^): 3085 (–NH–), 2945 (CH_Ar_), 1669 (–CO–NH–),1606 (–CH=N), 1061 (O–C); ^1^H-RMN (400 MHz, DMSO-*d6*): δ = 11.72 (s, 1H, CO–NH), 8.33/8.48 (s, 1H, CH=N), 8.05/8.07 (s, 1H, CH=N), 7.49–7.44/7.39–7.36 (m, 1H, Ar–H), 7.12 (d, *J* = 3.7 Hz, 2H, Ar–H), 5.53/5.11 (s, 2H, CH_2_–CO), 3.84 (s, 3H, O–CH_3_), 3.78/3.80 (s, 3H, O–CH_3_), 3.45 (s, 3H, N–CH_3_), 3.19 (s, 3H, N–CH_3_); ^13^C-NMR (101 MHz, DMSO-*d6*): δ = 168.8/163.2 (C_q_), 154.9 (C_q_), 153.1/153.0 (C_q_), 151.4 (C_q_), 148.4 (C_q_), 144.3/148.4 (CH=N), 144.0/144.2 (CH=N), 140.5/143.3 (CH_Ar_), 127.7/127.8 (C_q_), 124.8 (CH_Ar_), 117.2/117.4 (CH_Ar_), 114.7/114.8 (CH_Ar_), 107.1/106.8 (C_q_), 61.6 (O–CH_3_), 56.2 (O–CH_3_), 47.8/47.9 (N–CH_2_–CO), 29.9 (N–CH_3_), 27.8/27.9 (N–CH_3_); HRMS (EI-MS): *m/z* calculated for C_18_H_21_N_6_O_5_ [M + H]^+^ 401.15679, found 401.15654.

*N*-*(2,4*-*Dimethoxybenzylidene)*-*2*-*(1,3*-*dimethylxanthin*-*7*-*yl)acethydrazide* (**5i**) Tautomeric mixture (8:2). Yield 93%, white solid, m.p. >250 °C; IR (ATR diamond, cm^−1^): 3108 (–NH–), 2944 (CH_Ar_), 1658 (–CO–NH–), 1601 (–CH=N), 1135 (O–C); ^1^H-RMN (400 MHz, DMSO-*d6*): δ = 11.55/11.67 (s, 1H, CO–NH), 8.29/8.46 (s, 1H, CH=N), 8.04/8.06 (s, 1H, CH=N), 7.79 (d, *J* = 8.6 Hz, 1H, Ar–H)/7.70 (d, *J* = 8.5 Hz, 1H, Ar–H), 6.65–6.61 (m, 2H, Ar–H)/6.60 (d, 2H, Ar–H), 5.50/5.07 (s, 2H, CH_2_–CO), 3.85 (s, 3H, O–CH_3_), 3.82/3.81 (s, 3H, O–CH_3_), 3.45 (s, 3H, N–CH_3_), 3.19 (s, 3H, N–CH_3_); ^13^C-NMR (101 MHz, DMSO-*d6*): δ = 167.9/163.0 (C_q_), 162.8/162.7 (C_q_), 159.5/159.6 (C_q_), 154.9 (C_q_), 151.4 (C_q_), 148.2/148.4 (C_q_), 144.1/144.2 (CH=N), 140.4/143.4 (CH=N), 127.0/127.1 (CH_Ar_), 115.1 (C_q_), 107.1 (C_q_), 106.9/106.8 (CH_Ar_), 98.6/98.7 (CH_Ar_), 56.2 (O–CH_3_), 55.9 (O–CH_3_), 47.8/47.9 (N–CH_2_–CO), 29.9 (N–CH_3_), 27.8/27.9 (N–CH_3_); HRMS (EI-MS): *m/z* calculated for C_18_H_21_N_6_O_5_ [M + H]^+^ 401.15679, found 401.15669.

*N*-*(3,5*-*Dimethoxybenzylidene)*-*2*-*(1,3*-*dimethylxanthin*-*7*-*yl)acethydrazide* (**5j**) Tautomeric mixture (8:2). Yield 79%, white solid, m.p. >250 °C; IR (ATR diamond, cm^−1^): 3092 (–NH–), 2953 (CH_Ar_), 1662 (–CO–NH–), 1592 (–CH=N), 1052 (O–C); ^1^H-RMN (400 MHz, DMSO-*d6*): δ = 11.78 (s, 1H, CO–NH), 8.05/8.13 (s, 1H, CH=N), 7.96/8.06 (s, 1H, CH=N), 6.88/6.85 (d, *J* = 2.0 Hz, 2H, Ar–H), 6.56 (s, 1H, Ar–H), 5.54/5.12 (s, 2H, CH_2_–CO), 3.78/3.77 (s, 6H, O–CH_3_), 3.45/3.44 (s, 3H, N–CH_3_), 3.19 (s, 3H, N–CH_3_); ^13^C-NMR (101 MHz, DMSO-*d6*): δ = 168.4/163.3 (C_q_), 161.1/161.2 (C_q_), 154.8 (C_q_), 151.4 (C_q_), 148.3/148.4 (C_q_), 144.5/147.6 (CH=N), 144.0/144.1 (CH=N), 136.2/136.4 (C_q_), 107.1/106.8 (C_q_), 105.2/105.3 (CH_Ar_), 102.6/102.8 (CH_Ar_), 55.8 (O–CH_3_), 47.8/47.9 (N–CH_2_–CO), 29.9 (N–CH_3_), 27.8/27.9 (N–CH_3_); HRMS (EI-MS): *m/z* calculated for C_18_H_21_N_6_O_5_ [M + H]^+^ 401.15679, found 401.15661.

*N*-*(4*-*Methylbenzylidene)*-*2*-*(1,3*-*dimethylxanthin*-*7*-*yl)acethydrazide* (**5** **k**) Tautomeric mixture (8:2). Yield 91%, white solid, m.p. >250 °C; IR (ATR diamond, cm^−1^): 3109 (–NH–), 2964 (CH_Ar_), 1637 (–CO–NH–), 1544 (–CH=N); ^1^H-RMN (400 MHz, DMSO-*d6*): δ = 11.70 (s, 1H, CO–NH), 8.05/8.18 (s, 1H, CH=N), 8.01/8.07 (s, 1H, CH=N), 7.62/7.58 (d, *J* = 7.9 Hz, 2H, Ar–H), 7.27 (d, *J* = 7.7 Hz, 2H, Ar–H)/7.27 (d, *J* = 7.7 Hz, 1H, Ar–H) and 7.24 (s, 1H, Ar–H), 5.54/5.11 (s, 2H, CH_2_–CO), 3.46 (s, 3H, N–CH_3_), 3.20 (s, 3H, N–CH_3_), 2.34 (s, 3H, N–CH_3_); ^13^C-NMR (101 MHz, DMSO-*d6*): δ = 168.2/163.1 (C_q_), 154.9 (C_q_), 151.4 (C_q_), 148.3/148.4 (C_q_), 144.8/147.8 (CH=N), 144.1/144.2 (CH=N), 140.4/140.5 (C_q_), 131.6/131.7 (C_q_), 129.9 (CH_Ar_), 127.3/127.5 (CH_Ar_), 107.1/106.8 (C_q_), 47.8/47.9 (N–CH_2_–CO), 29.9 (N-CH_3_), 27.8/27.9 (N-CH_3_), 21.4 (Ar–CH_3_); HRMS (EI-MS): *m/z* calculated for C_17_H_19_N_6_O_3_ [M + H]^+^ 355.15131, found 355.15115.

##### Synthesis of the theophyllinyl-acetamido-thiazolidin-4-one derivatives (**6a–k**)

Hydrazide derivatives (**5a–k**) (5 mmol) were reacted with thioglycolic acid (100 mmol) using freshly distillated toluene as solvent, according to the procedure described for other thiazolidine-4-one derivatives [35]. The mixture was heated under reflux and stirred at 120 °C for 18 h. The reaction was monitored by Thin Layer Chromatography (TLC), in UV light at 254 nm, using ethyl acetate: methanol (9.6:0.4, v/v) as eluent system. At the end of the reaction, the solvent was removed and the mixture was cooled at 0 °C on ice bath. After that, dichloromethane (100 mL) was added and the mixture was neutralized, under continuous stirring at 0 °C, with sodium bicarbonate 10%. The organic layer was separated and washed with alkaline solution (two times with 100 mL) and then acidulated with hydrochloric acid 10% (300 mL). Finally, the organic phase was dried on anhydrous MgSO_4_, and it was concentrated by rotary evaporator under reduce pressure. The residue was purified on silica gel column, using ethyl acetate as eluent solvent.

*2*-*Phenyl*-*3*-*[(1,3*-*dimethylxanthin*-*7*-*yl)acetamido]thiazolidine*-*4*-*one* (**6a**) Yield 50%, white solid, m.p. 251–252 °C; IR (ATR diamond, cm^−1^): 3022 (–NH–), 2926 (CH_Ar_), 1694 (C=O), 1686 (–CO–NH–), 699(C-S); ^1^H-RMN (400 MHz, CDCl_3_): δ = 9.50 (s, 1H, CO–NH), 7.63 (s, 1H, N–CH–N), 7.21–7.07 (m, 5H, Ar–H), 5.79 (s, 1H, N–CH–S), 4.96 (d, J = 14.0 Hz, 1H, CH_2_–CO), 4.54 (d, J = 14.0 Hz, 1H, CH_2_–CO), 3.80 (d, J = 16.0 Hz, 1H, CH_2_–S), 3.67 (d, J = 16.0 Hz, 1H, CH_2_–S), 3.60 (s, 3H, N–CH_3_), 3.22 (s, 3H, N–CH_3_); ^13^C-NMR (101 MHz, CDCl_3_): δ = 168.9 (C_q_), 163.7 (C_q_), 155.9 (C_q_), 150.9 (C_q_), 149.1 (C_q_), 141.7 (CH=N), 136.2 (C_q_), 129.2 (CH_Ar_), 128.2 (CH_Ar_), 128.0 (CH_Ar_), 106.0 (C_q_), 61.6 (N–CH–S), 48.8 (N–CH_2_–CO), 30.02 (S–CH_2_–CO), 29.8 (N–CH_3_), 28.1 (N-CH_3_); HRMS (EI-MS): *m/z* calculated for C_18_H_19_N_6_O_4_S [M + H]^+^ 415.11830, found 415.11817.

*2*-*(4*-*Bromophenyl)*-*3*-*[(1,3*-*dimethylxanthin*-*7*-*yl)acetamido]thiazolidine*-*4*-*one* (**6b**) Yield 30%, white solid, m.p. >250 °C; IR (ATR diamond, cm^−1^): 3106 (–NH–), 3014 (CH_Ar_), 1698 (C=O), 1659 (–CO–NH–), 812 (C–Br), 680 (C–S); ^1^H-RMN (400 MHz, CDCl_3_): δ = 9.58 (s, 1H, CO–NH), 7.64 (s, 1H, N–CH–N), 7.30 (d, J = 8.1 Hz, 2H, Ar–H), 7.08 (d, J = 8.1 Hz, 2H, Ar–H), 5.79 (s, 1H, N–CH–S), 4.89 (d, J = 14.0 Hz, 1H, CH_2_–CO), 4.60 (d, J = 14.1 Hz, 1H, CH_2_–CO), 3.78 (d, J = 16.0 Hz, 1H, CH_2_–S), 3.67 (d, J = 16.0 Hz, 1H, CH_2_–S), 3.63 (s, 3H, N–CH_3_), 3.31 (s, 3H, N–CH_3_); ^13^C-NMR (101 MHz, CDCl_3_): δ = 168.8 (C_q_), 163.8 (C_q_), 156.0 (C_q_), 150.5 (C_q_), 149.3 (C_q_), 141.9 (CH=N), 131.5 (CH_Ar_), 131.3 (C_q_), 129.7 (CH_Ar_), 123.7 (C_q_), 106.1 (C_q_), 61.3 (N–CH–S), 48.8 (N–CH_2_–CO), 30.1 (S–CH_2_–CO), 29.9 (N–CH_3_), 28.1 (N–CH_3_); HRMS (EI-MS): *m/z* calculated for C_18_H_18_BrN_6_O_4_S [M + H]^+^ 493.02881, found 493.02831.

*2*-*(4*-*Chlorophenyl)*-*3*-*[(1,3*-*dimethylxanthin*-*7*-*yl)acetamido]thiazolidine*-*4*-*one* (**6c**) Yield 29%, white solid, m.p. >250 °C; IR (ATR diamond, cm^−1^): 3111 (–NH–), 2972 (CH_Ar_), 1698 (C=O), 1658 (–CO–NH–), 826 (C–Cl), 679 (C–S); ^1^H-RMN (250 MHz, CDCl_3_): δ = 9.58 (s, 1H, CO–NH), 7.64 (s, 1H, N–CH-N), 7.13 (s, 4H, Ar–H), 5.81 (s, 1H, N–CH–S), 4.90 (d, J = 14.1 Hz, 1H, CH_2_–CO), 4.59 (d, J = 14.1 Hz, 1H, CH_2_–CO), 3.79 (dd, J = 16.0 Hz, 1.2 Hz, 1H, CH_2_–S), 3.71–3.63 (m, 1H, CH_2_–S), 3.62 (s, 3H, N–CH_3_), 3.30 (s, 3H, N–CH_3_); ^13^C-NMR (63 MHz, CDCl_3_): δ = 168.7 (C_q_), 163.8 (C_q_), 156.0 (C_q_), 150.7 (C_q_), 149.3 (C_q_), 141.9 (CH=N), 135.6 (C_q_), 134.6 (C_q_), 129.5 (CH_Ar_), 128.5 (CH_Ar_), 106.1 (C_q_), 61.1 (N–CH–S), 48.8 (N–CH_2_–CO), 30.0 (S–CH_2_–CO), 29.9 (N–CH_3_), 28.0 (N-CH_3_); HRMS (EI-MS): *m/z* calculated for C_18_H_18_ClN_6_O_4_S [M + H]^+^ 449.07932, found 449.07900.

*2*-*(4*-*Fluorophenyl)*-*3*-*[(1,3*-*dimethylxanthin*-*7*-*yl)acetamido]thiazolidine*-*4*-*one* (**6d**) Yield 28%, white solid, m.p. 262 °C; IR (ATR diamond, cm^−1^): 3116 (–NH–), 2991 (CH_Ar_), 1697 (C=O), 1659 (–CO–NH–), 1224 (C–F), 678 (C–S); ^1^H-RMN (400 MHz, CDCl_3_): δ = 9.51 (s, 1H, CO–NH), 7.63 (s, 1H, N–CH–N), 7.21–7.15 (m, 2H, Ar–H), 6.82 (t, J = 8.3 Hz, 2H, Ar–H), 5.83 (s, 1H, N–CH–S), 4.91 (d, J = 14.0 Hz, 1H, CH_2_–CO), 4.58 (d, J = 14.1 Hz, 1H, CH_2_–CO), 3.79 (d, J = 16.0 Hz, 1H, CH_2_–S), 3.67 (d, J = 16.2 Hz, 1H, CH_2_–S), 3.61 (s, 3H, N–CH_3_), 3.30 (s, 3H, N–CH_3_); ^13^C-NMR (63 MHz, CDCl_3_): δ = 168.6 (C_q_), 164.3 (C_q_), 163.7 (C_q_), 156.0 (C_q_), 150.7 (C_q_), 149.3 (CH=N), 141.8 (C_q_), 131.9 (C_q_), 130.2 (CH_Ar_), 130.1 (CH_Ar_), 115.4 (CH_Ar_), 115.2 (CH_Ar_), 106.1 (C_q_), 61.1 (N–CH–S), 48.8 (N–CH_2_–CO), 30.0 (S–CH_2_–CO), 29.9 (N–CH_3_), 28.0 (N–CH_3_); ^19^F-NMR (376 MHz, CDCl_3_, δppm): −110.2 (s, 1F, Ar–F); HRMS (EI-MS): *m/z* calculated for C_18_H_18_FN_6_O_4_S [M + H]^+^ 433.10887, found 433.10866.

*2*-*(2*-*Methoxyphenyl)*-*3*-*[(1,3*-*dimethylxanthin*-*7*-*yl)acetamido]thiazolidine*-*4*-*one* (**6e**) Yield 33%, white powder, m.p. 209–210 °C; IR (ATR diamond, cm^−1^): 3093 (–NH–), 2991 (CH_Ar_), 1683 (C=O), 1654 (–CO–NH–), 1107 (O–C), 696 (C–S); ^1^H-RMN (250 MHz, CDCl_3_): δ = 9.46 (s, 1H, CO–NH), 7.64 (s, 1H, N–CH–N), 7.20–7.11 (m, 1H, Ar–H), 7.07 (dd, J = 7.6, 1.5 Hz, 1H, Ar–H), 6.73 (t, J = 7.5 Hz, 1H, Ar–H), 6.66 (d, J = 8.2 Hz, 1H, Ar–H), 6.10 (s, 1H, N–CH–S), 4.96 (d, J = 14.2 Hz, 1H, CH_2_–CO), 4.61 (d, J = 14.2 Hz, 1H, CH_2_–CO), 3.70 (s, 3H, O–CH_3_), (s, 2H, CH_2_–S), 3.58 (s, 3H, N–CH_3_), 3.24 (s, 3H, N–CH_3_); ^13^C-NMR (63 MHz, CDCl_3_): δ = 169.7 (C_q_), 163.6 (C_q_), 157.5 (C_q_), 155.9 (C_q_), 150.9 (C_q_), 149.0 (C_q_), 141.8 (CH=N), 130.1 (CH_Ar_), 128.9 (CH_Ar_), 124.7 (C_q_), 120.2 (CH_Ar_), 110.3 (CH_Ar_), 106.1 (C_q_), 57.0 (N–CH–S), 55.5 (O–CH_3_), 48.7 (N–CH_2_–CO), 30.0 (S–CH_2_–CO), 29.8 (N–CH_3_), 28.1 (N–CH_3_); HRMS (EI-MS): *m/z* calculated for C_19_H_21_N_6_O_5_S [M + H]^+^ 445.12886, found 445.12838.

*2*-*(4*-*Methoxyphenyl)*-*3*-*[(1,3*-*dimethylxanthin*-*7*-*yl)acetamido]thiazolidine*-*4*-*one* (**6f**) Yield 37%, white powder, m.p. 239–240 °C; IR (ATR diamond, cm^−1^): 3106 (–NH–), 2961 (CH_Ar_), 1697 (C=O), 1657 (–CO–NH–), 1114 (O–C), 680(C–S); ^1^H-RMN (400 MHz, CDCl_3_): δ = 9.57 (s, 1H, CO–NH), 7.63 (s, 1H, N–CH–N), 7.06 (d, J = 8.1 Hz, 2H, Ar–H), 6.60 (d, J = 8.1 Hz, 2H, Ar–H), 5.77 (s, 1H, N–CH–S), 4.94 (d, J = 14.0 Hz, 1H, CH_2_–CO), 4.54 (d, J = 14.0 Hz, 1H, CH_2_–CO), 3.78 (d, J = 17.1 Hz, 1H, CH_2_–S), 3.75 (s, 3H, O–CH_3_), 3.65 (d, J = 16.0 Hz, 1H, CH_2_–S), 3.60 (s, 3H, N–CH_3_), 3.25 (s, 3H, N–CH_3_); ^13^C-NMR (101 MHz, CDCl_3_): δ = 168.8 (C_q_), 163.6 (C_q_), 160.4 (C_q_), 156.0 (C_q_), 150.7 (C_q_), 149.2 (C_q_), 141.7 (CH=N), 129.5 (CH_Ar_), 127.4 (C_q_), 113.4 (CH_Ar_), 106.1 (C_q_), 61.4 (N–CH–S), 55.2 (O–CH_3_), 48.9 (N–CH_2_–CO), 30.1 (S–CH_2_–CO), 29.9 (N–CH_3_), 28.0 (N–CH_3_); HRMS (EI-MS): *m/z* calculated for C_19_H_21_N_6_O_5_S [M + H]^+^ 445.12886, found 445.128533.

*2*-*(3*-*Methoxyphenyl)*-*3*-*[(1,3*-*dimethylxanthin*-*7*-*yl)acetamido]thiazolidine*-*4*-*one* (**6** **g**) Yield 50%, white solid, m.p. 199–200 °C; IR (ATR diamond, cm^−1^): 3013 (–NH–), 2951 (CH_Ar_), 1701 (C=O), 1654 (–CO–NH–), 1175 (O–C), 694 (C–S); ^1^H-RMN (400 MHz, CDCl_3_): δ = 9.56(s, 1H, CO–NH), 7.64 (s, 1H, N–CH–N), 7.00 (t, J = 7.9 Hz, 1H, Ar–H), 6.70 (d, J = 7.6 Hz, 2H, Ar–H), 6.62 (s, 1H, Ar–H), 5.75 (s, 1H, N–CH–S), 4.99 (d, J = 14.0 Hz, 1H, CH_2_–CO), 4.53 (d, J = 14.0 Hz, 1H, CH_2_–CO), 3.80 (d, J = 16.0 Hz, 1H, CH_2_–S), 3.70 (s, 3H, O–CH_3_), 3.67 (d, J = 16.2 Hz, 1H, CH_2_–S), 3.60 (s, 3H, N–CH_3_), 3.23 (s, 3H, N–CH_3_); ^13^C-NMR (101 MHz, CDCl_3_): δ = 168.9 (C_q_), 163.7 (C_q_), 157.6 (C_q_), 156.0 (C_q_), 150.9 (C_q_), 149.1 (C_q_), 141.6 (CH=N), 137.6 (C_q_), 129.2 (CH_Ar_), 120.1 (CH_Ar_), 115.3 (CH_Ar_), 112.6 (CH_Ar_), 106.0 (C_q_), 61.6 (N–CH–S), 55.2 (O–CH_3_), 48.9 (N–CH_2_–CO), 30.0 (S–CH_2_–CO), 29.8 (N–CH_3_), 28.0 (N–CH_3_); HRMS (EI-MS): *m/z* calculated for C_19_H_21_N_6_O_5_S [M + H]^+^ 445.12886, found 445.12836.

*2*-*(2,3*-*Dimethoxyphenyl)*-*3*-*[(1,3*-*dimethylxanthin*-*7*-*yl)acetamido]thiazolidine*-*4*-*one* (**6h**) Yield 50%, white powder, m.p. 247–248 °C; IR (ATR diamond, cm^−1^): 3110 (–NH–), 2983 (CH_Ar_), 1695 (C=O), 1651 (–CO–NH–), 1175 (O–C), 692 (C–S); ^1^H-RMN (400 MHz, CDCl_3_): δ = 9.52 (s, 1H, CO–NH), 7.64 (s, 1H, N–CH–N), 6.91 (t, J = 7.9 Hz, 1H, Ar–H), 6.81 (d, J = 7.8 Hz, 1H, Ar–H), 6.76 (d, J = 8.1 Hz, 1H, Ar–H), 6.09 (s, 1H, N–CH–S), 4.99 (d, J = 14.0 Hz, 1H, CH_2_–CO), 4.59 (d, J = 14.1 Hz, 1H, CH_2_–CO), 3.80 (s, 3H, O–CH_3_), 3.72 (d, J = 9.1 Hz, 2H, CH_2_–S), 3.59 (s, 6H, O–CH_3_, N–CH_3_), 3.25 (s, 3H, N–CH_3_); ^13^C-NMR (101 MHz, CDCl_3_): δ = 169.34 (C_q_), 163.54 (C_q_), 156.06 (C_q_), 152.31 (C_q_), 151.05 (C_q_), 149.21 (C_q_), 147.6 (C_q_), 141.76 (CH=N), 130.03 (C_q_), 123.83 (CH_Ar_), 120.02 (CH_Ar_), 112.71 (CH_Ar_), 106.26 (C_q_), 61.00 (N–CH–S), 56.20 (O–CH_3_), 55.87 (O–CH_3_), 48.94 (N–CH_2_–CO), 29.98 (S–CH_2_–CO), 29.83 (N–CH_3_), 28.13 (N–CH_3_); HRMS (EI-MS): *m/z* calculated for C_20_H_23_N_6_O_6_S [M + H]^+^ 475.13943, found 475.139544.

*2*-*(2,4*-*Dimethoxyphenyl)*-*3*-*[(1,3*-*dimethylxanthin*-*7*-*yl)acetamido]thiazolidine*-*4*-*one* (**6i**) Yield 6%, yellow powder, m.p. >250 °C; IR (ATR diamond, cm^−1^): 3113 (–NH–), 2966 (CH_Ar_), 1684 (C=O), 1616 (–CO–NH–), 1022 (O–C), 665 (C–S); ^1^H-RMN (400 MHz, CDCl_3_): δ = 9.50 (s, 1H, CO–NH), 7.64 (s, 1H, CH=N), 6.99 (d, J = 8.4 Hz, 1H, Ar–H), 6.26–6.21 (m, 1H, Ar–H), 6.19 (s, 1H, Ar–H), 6.06 (s, 1H, N–CH–S), 4.95 (d, J = 14,3 Hz, 1H, CH_2_–CO), 4.61 (d, J = 14.3 Hz, 1H, CH_2_–CO), 3.74 (s, 3H, O–CH_3_), 3.71–3.61 (m, 5H, CH_2_–S, O–CH_3_), 3.58 (s, 3H, N–CH_3_), 3.26 (s, 3H, N–CH_3_); ^13^C-NMR (101 MHz, CDCl_3_): δ = 169.6 (C_q_), 163.6 (C_q_), 161.6 (C_q_), 158.8 (C_q_), 155.9 (C_q_), 150.8 (C_q_), 149.0 (C_q_), 141.9 (CH=N), 130.0 (CH_Ar_), 116.6 (C_q_), 106.1 (C_q_), 104.0 (CH_Ar_), 97.9 (CH_Ar_), 56.9 (N–CH–S), 55.5 (O–CH_3_), 55.3 (O–CH_3_), 48.7 (N–CH_2_–CO), 30.1 (S–CH_2_–CO), 29.8 (N–CH_3_), 28.0 (N–CH_3_); HRMS (EI-MS): *m/z* calculated for C_20_H_23_N_6_O_6_S [M + H]^+^ 475.13943, found 475.13968.

*2*-*(3,5*-*Dimethoxyphenyl)*-*3*-*[(1,3*-*dimethylxanthin*-*7*-*yl)acetamido]thiazolidine*-*4*-*one* (**6j**) Yield 54%, white solid, m.p. 247–248 °C; IR (ATR diamond, cm^−1^): 3045 (-NH-), 2994 (CH_Ar_), 1682 (C=O), 1655 (–CO–NH–), 1052 (O-C), 671 (C-S); ^1^H-RMN (400 MHz, CDCl_3_): δ = 9.65 (s, 1H, CO–NH), 7.64 (s, 1H, N–CH–N), 6.23 (s, 3H, Ar–H), 5.69 (s, 1H, N–CH–S), 5.02 (d, J = 13.9 Hz, 1H, CH_2_–CO), 4.52 (d, J = 13.9 Hz, 1H, CH_2_–CO), 3.79 (d, J = 15.9 Hz, 1H, CH_2_–S), 3.68 (s, 6H, O–CH_3_), 3.64 (s, CH_2_–S), 3.59 (s, 3H, N–CH_3_), 3.24 (s, 3H, N–CH_3_); ^13^C-NMR (101 MHz, CDCl_3_): δ = 169.03 (C_q_), 163.7 (C_q_), 160.8 (C_q_), 156.0 (C_q_), 150.9 (C_q_), 149.1 (C_q_), 141.5 (CH=N), 138.3 (C_q_), 106.0 (C_q_), 105.2 (CH_Ar_), 101.6 (CH_Ar_), 61.7 (N–CH–S), 55.3 (O–CH_3_), 48.9 (N–CH_2_–CO), 30.0 (S–CH_2_–CO), 29.8 (N–CH_3_), 27.9 (N–CH_3_); HRMS (EI-MS): *m/z* calculated for C_20_H_23_N_6_O_6_S [M + H]^+^ 475.13943, found 475.13967.

*2*-*(4*-*Methylphenyl)*-*3*-*[(1,3*-*dimethylxanthin*-*7*-*yl)acetamido]thiazolidine*-*4*-*one* (**6** **k**) Yield 52%, white powder, m.p. >250; IR (ATR diamond, cm^−1^): 3106 (–NH–), 2920 (CH_Ar_), 1696 (C=O), 1656 (–CO–NH–), 1021 (O–C), 680 (C–S); ^1^H-RMN (400 MHz, CDCl_3_): δ = 9.54 (s, 1H, CO–NH), 7.64 (s, 1H, N–CH–N), 7.02 (d, J = 7.7 Hz, 2H, Ar–H), 6.93 (d, J = 7.8 Hz, 2H, Ar–H), 5.73 (s, 1H, N–CH–S), 4.95 (d, J = 14.0 Hz, 1H, CH_2_–CO), 4.56 (d, J = 14.1 Hz, 1H, CH_2_–CO), 3.78 (d, J = 16.0 Hz, 1H, CH_2_–S), 3.67 (d, J = 16.1 Hz, 1H, CH_2_–S), 3.60 (s, 3H, N–CH_3_), 3.23 (s, 3H, N–CH_3_), 2.28 (s, 3H, Ar–CH_3_); ^13^C-NMR (101 MHz, CDCl_3_): δ = 169.0 (C_q_), 163.6 (C_q_), 156.0 (C_q_), 150.8 (C_q_), 149.1 (C_q_), 141.8 (C_q_), 139.8 (CH=N), 133.1 (C_q_), 128.9 (CH_Ar_), 128.0 (CH_Ar_), 106.2 (C_q_), 61.6 (N–CH–S), 48.9 (N–CH_2_–CO), 30.0 (S–CH_2_–CO), 29.9 (N–CH_3_), 28.0 (N–CH_3_), 21.2 (Ar–CH_3_); HRMS (EI-MS): *m/z* calculated for C_19_H_21_N_6_O_4_S [M + H]^+^ 429.13395, found 429.13383 (Additional file 1).

#### Biological evaluation

The antioxidant activity was estimated using in vitro tests: DPPH and ABTS radical scavenging ability and total antioxidant capacity.

##### DPPH radical scavenging assay

The antiradical activity against 1,1-diphenyl-2-picrylhydrazyl radical (DPPH) was measured as described in [32] with slight modifications. Different samples of the tested compounds (50, 100, 150, 200, 250 μL) from a stock solution (20 mg/mL in DMSO) were mixed with DMSO to obtain 1300 μL, and then DPPH in methanol (1200 μL, 15 μM) was added. The final concentrations of the compound in the test tube were 0.4, 0.8, 1.2, 1.6, and 2 mg/mL, respectively. The mixture was left for 30 min in the darkness and, after that, the absorbance was measured at 517 nm against a blank solution (methanol). The radical scavenging capacity was calculated using the following equation:

$$\begin{aligned} {\text{Inhibition percent }}\% &= \left( {{\text{A}}_{\text{control}} - {\text{A}}_{\text{sample}} /{\text{A}}_{\text{control}} } \right) \\ & \quad \times 100. \end{aligned}$$

where A_control_ is the absorbance of the mixture of 1300 μL DMSO and 1200 μL DPPH after 30 min; A_sample_ is the absorbance of the sample after 30 min.

For the most active compounds, the effective concentration (EC_50_) was calculated by linear regression analysis. The theophylline (parent compound) and ascorbic acid (vitamin C) were used as reference and positive control, respectively. All tests were performed in triplicate.

##### ABTS radical scavenging assay

ABTS^+^ radicals were activated by reacting of ABTS (2,2′-azinobis(3-ethylbenzthiazoline-6-sulphonic acid)) (7 mM) with ammonium persulphate (2.45 mM) and the mixture was left for 16 h in the darkness, at room temperature. The resulted ABTS^·+^ solution was diluted with ethanol to obtain an absorbance value of 0.7 ± 0.02 at 734 nm as described in [33]. Different samples of the tested compounds (50, 100, 150, 200 μL) from a stock solution (10 mg/mL in DMSO) were mixed with DMSO to obtain 200 μL, and then freshly ABTS^·+^ solution (1800 μL) was added. The final concentrations of the compound in the test tube were 0.25, 0.50, 0.75 and 1 mg/mL, respectively. The mixture was stirred and left in the dark for 6 min., at room temperature, and the absorbance was measured at 734 nm. The radical scavenging capacity was calculated according to the following equation:

$$\begin{aligned} {\text{Scavenging activity }}\% &= \left( {{\text{A}}_{\text{control}} - {\text{A}}_{\text{sample}} /{\text{A}}_{\text{control}} } \right) \\ & \quad \times 100. \end{aligned}$$

where A_control_ is the absorbance of the mixture of 200 μL DMSO and 1800 μL ABTS^·+^ after 6 min; A_sample_ is the absorbance of sample after 6 min.

For the most active compounds the effective concentration (EC_50_) was calculated by linear regression analysis. The theophylline (parent compound) and ascorbic acid (vitamin C) were used as reference and positive control, respectively. All tests were performed in triplicate.

##### Phosphomolybdenum reducing antioxidant power (PRAP) assay

The phosphomolybdenum method was used according to the procedure described in [34] with few modifications. Different samples of the tested compounds (25, 50, 75, 100, 150 μL) from a stock solution (2.5 mg/mL in DMSO) were mixed with DMSO to obtain 150 μL, and then 2 mL of reagent solution (0.6 M sulfuric acid, 28 mM sodium phosphate, 4 mM ammonium molybdate) were added. The test tubes were closed and incubated in the oven at 95 °C, for 90 min. After cooling at room temperature, the absorbance was measured at 695 nm against a blank solution (DMSO with 2 mL reagent solution). The final concentrations of the compound in the test tube were 0.0291, 0.0582, 0.0872, 0.1163 and 0.1745 mg/mL. EC_50_ was calculated for all the tested compounds. Theophylline (parent compound) and ascorbic acid (vitamin C) were used as reference and positive control, respectively. All tests were performed in triplicate.

##### Statistical analysis

All antioxidant assays were carried out in triplicate. Data were analyzed by an analysis of variance (ANOVA) (p < 0.05) and were expressed by mean ± SD. The EC_50_ values were calculated by linear interpolation between the values registered above and below 50% activity.

## Conclusions

In this research new xanthine derivatives based on thiazolidine-4-one scaffold have been synthetized. The intermediate and final compounds were characterized in terms of physical properties and their structure was proved using spectral methods (FT-IR, ^1^H-NMR, ^13^C-NMR, ^19^F-NMR, HRMS). The antioxidant potential was evaluated using in vitro assays: DPPH and ABTS radical scavenging ability and phosphomolybdenum reducing antioxidant power assays. Some of the thiazolidin-4-one derivatives showed improved antioxidant effects in comparison to the corresponding hydrazones and parent compound, theophylline. A good antiradical scavenging effect (DPPH, ABTS) was shown by **6f** (R=4-OCH_3_), **6d** (4-F), **6c** (4-Cl) and **6k** (4-CH_3_). The compounds **6c** and **6k** showed also a high phosphomolybdenum reducing antioxidant power. The preliminary results support the antioxidant potential of some thiazolidine-4-one derivatives and motivate our next research focused on streptozotocin-induced diabetic rat model.

## Additional file

**Additional file 1.** NMR and HRMS spectra for thiazolidine-4-one derivatives (**6a–k**).
