# Supplementary material for: Synthesis and biological evaluation of the new 1,3-dimethylxanthine derivatives with thiazolidine-4-one scaffold
Source: Chem Cent J. 2017 Feb 1;11:12. doi: 10.1186/s13065-017-0241-0 (PMC5289128; doi:10.1186/s13065-017-0241-0)
Supplement: Supplementary file 1 — Additional file 1. NMR and HRMS spectra for thiazolidine-4-one derivatives (6a–k). [file 13065_2017_241_MOESM1_ESM.pdf]

NMR AND HRMS SPECTRA FOR THIAZOLIDINE-4-ONE DERIVATIVES (**6a-k**)

2-Phenyl-3-[(1,3-dimethylxanthin-7-yl)acethamido]thiazolidine-4-one (**6a**)

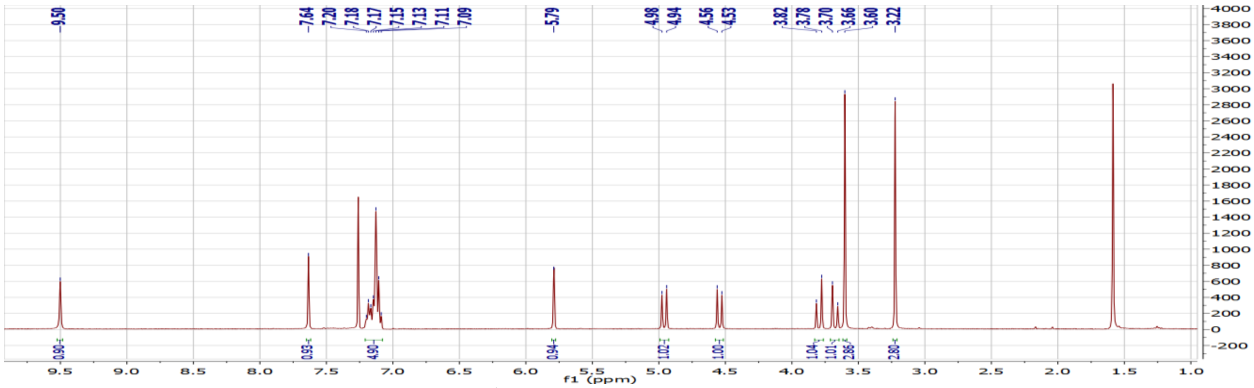

^{1}H-RMN spectra of **6a**

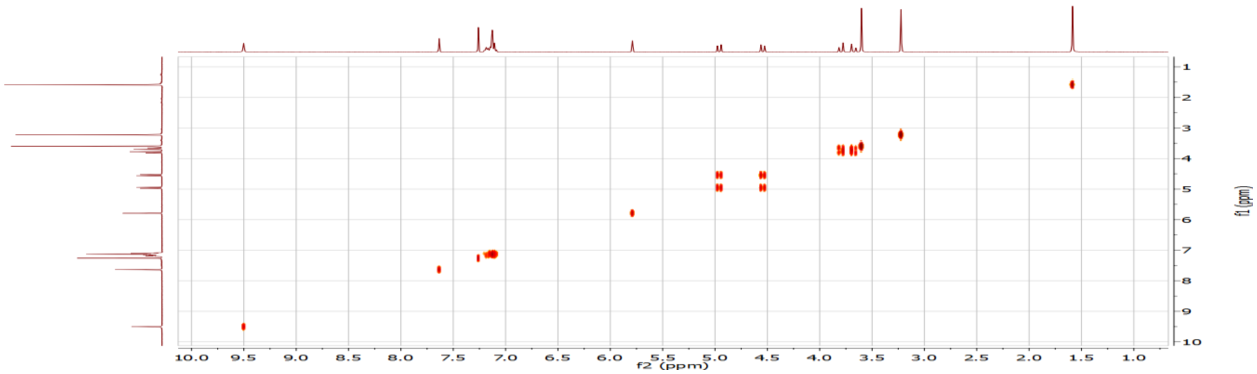

2D-COSY spectra of **6a**

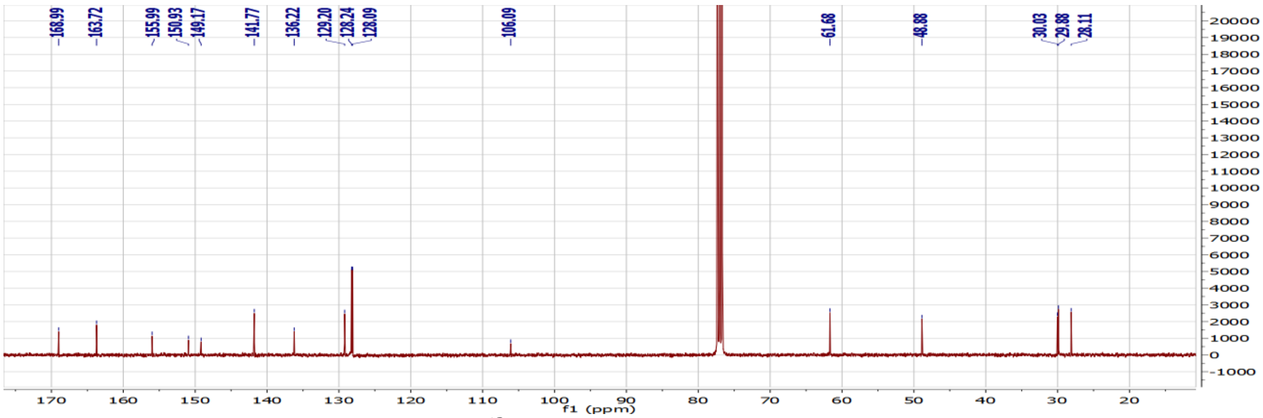

^{13}C-RMN spectra of **6a**

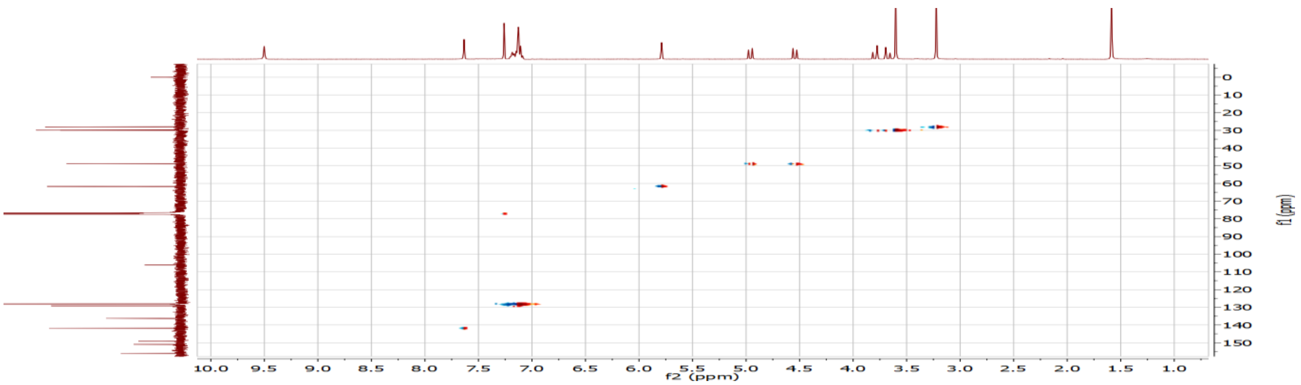

HSQC spectra of **6a**

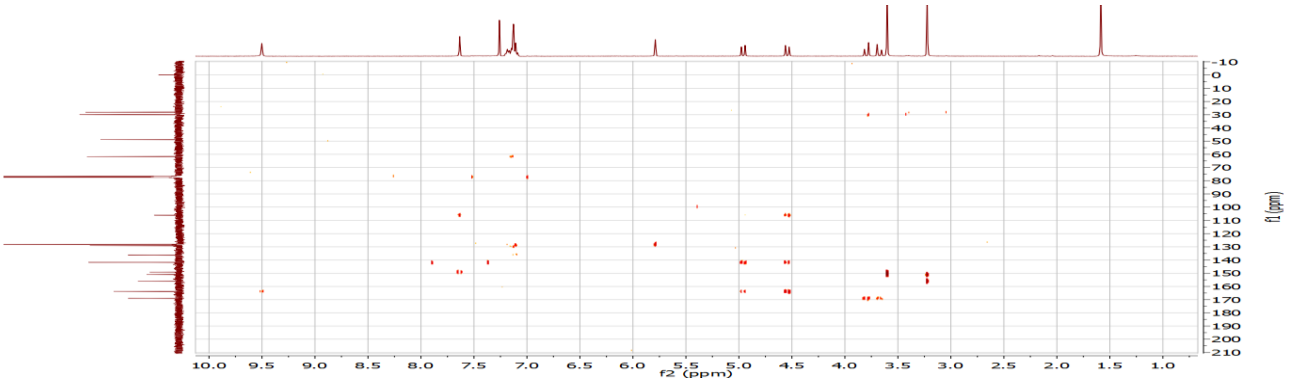

HMBC spectra of **6a**

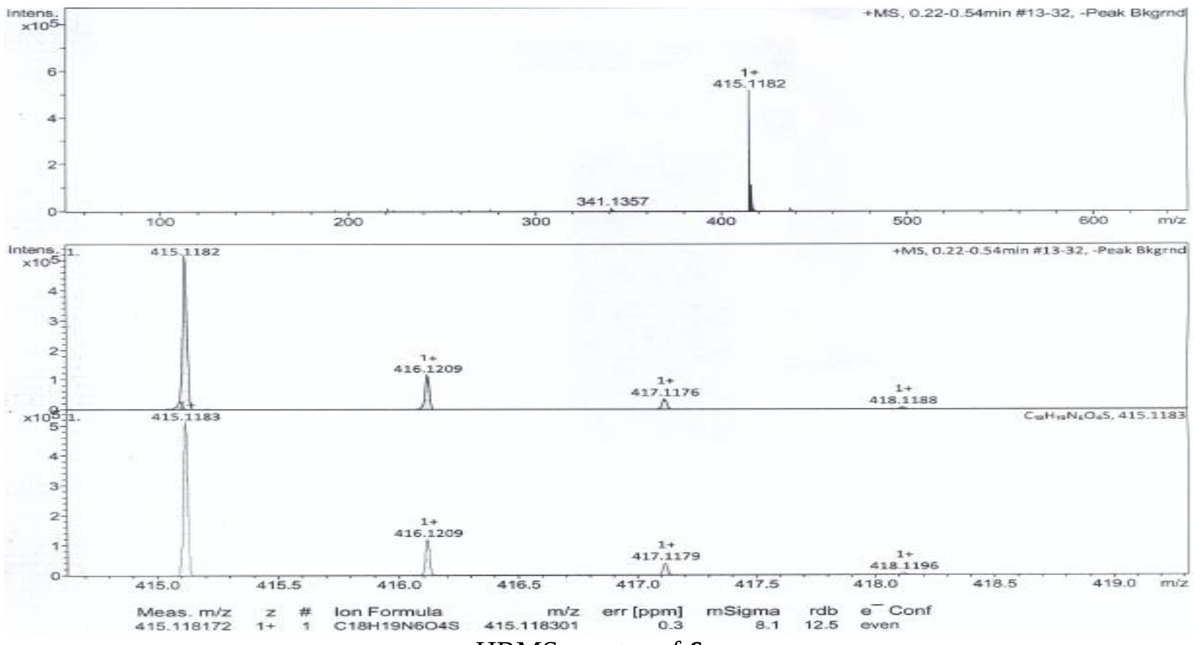

HRMS spectra of **6a**

2-(4-Bromophenyl)-3-[(1,3-dimethylxanthin-7-yl)acethamido]thiazolidine-4-one (**6b**)

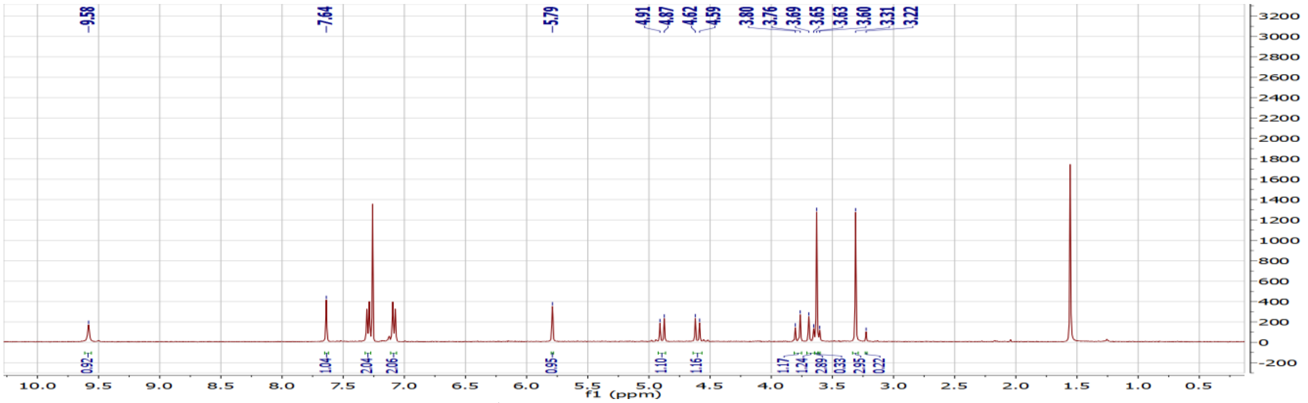

^{1}H-RMNspectra of **6b**

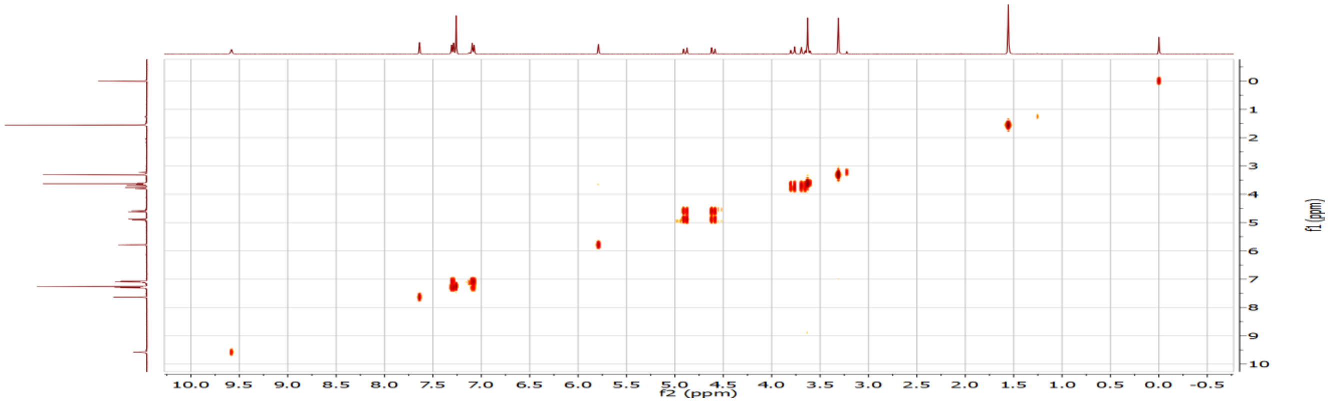

2D-COSYspectra of **6b**

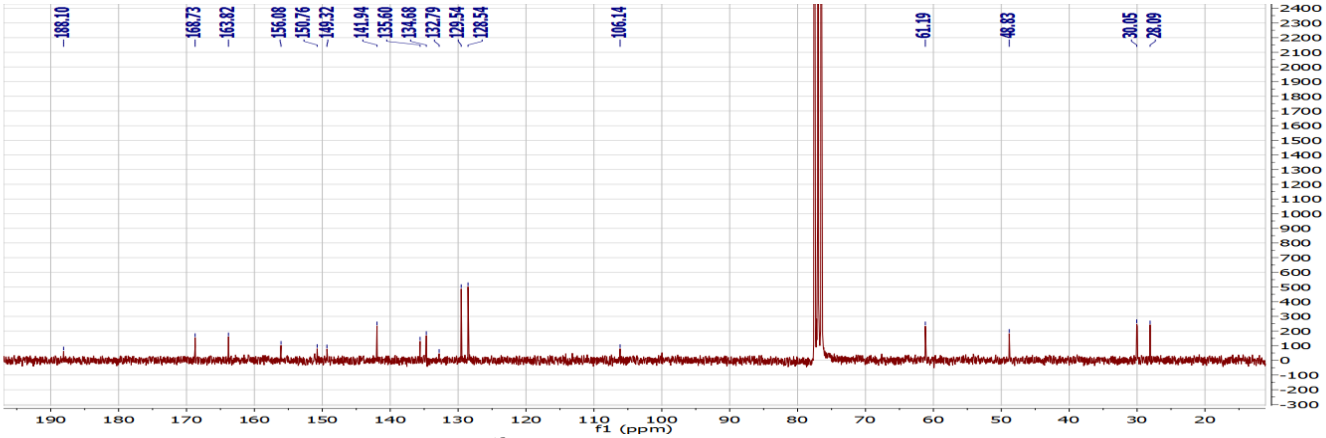

^{13}C-RMNspectra of **6b**

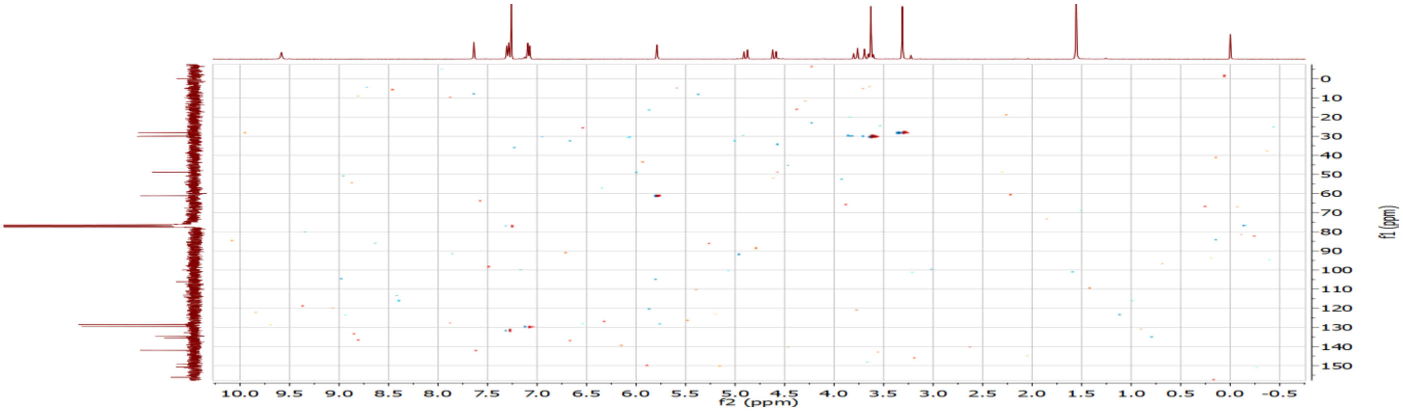

HSQC spectra of **6b**

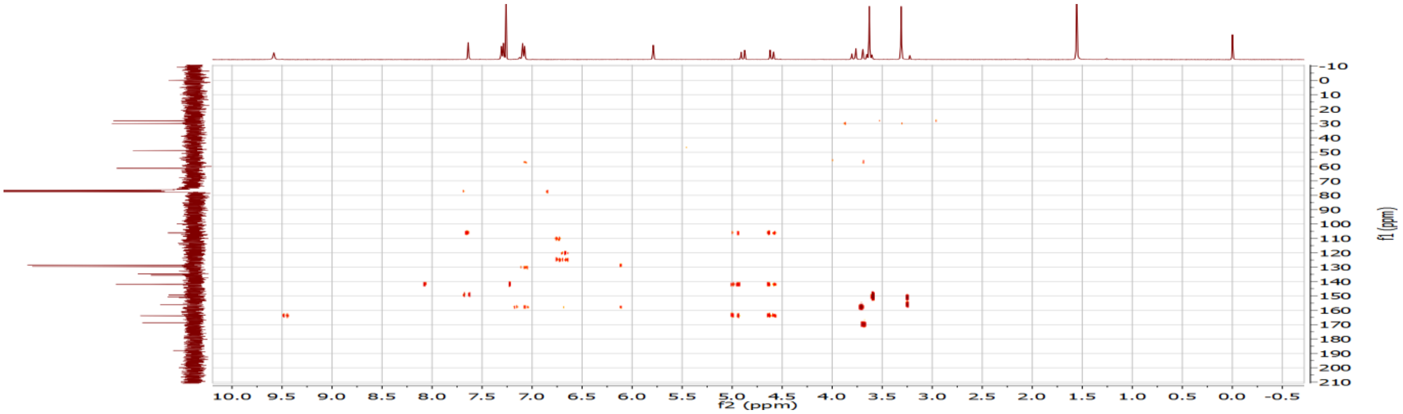

HMBC spectra of **6b**

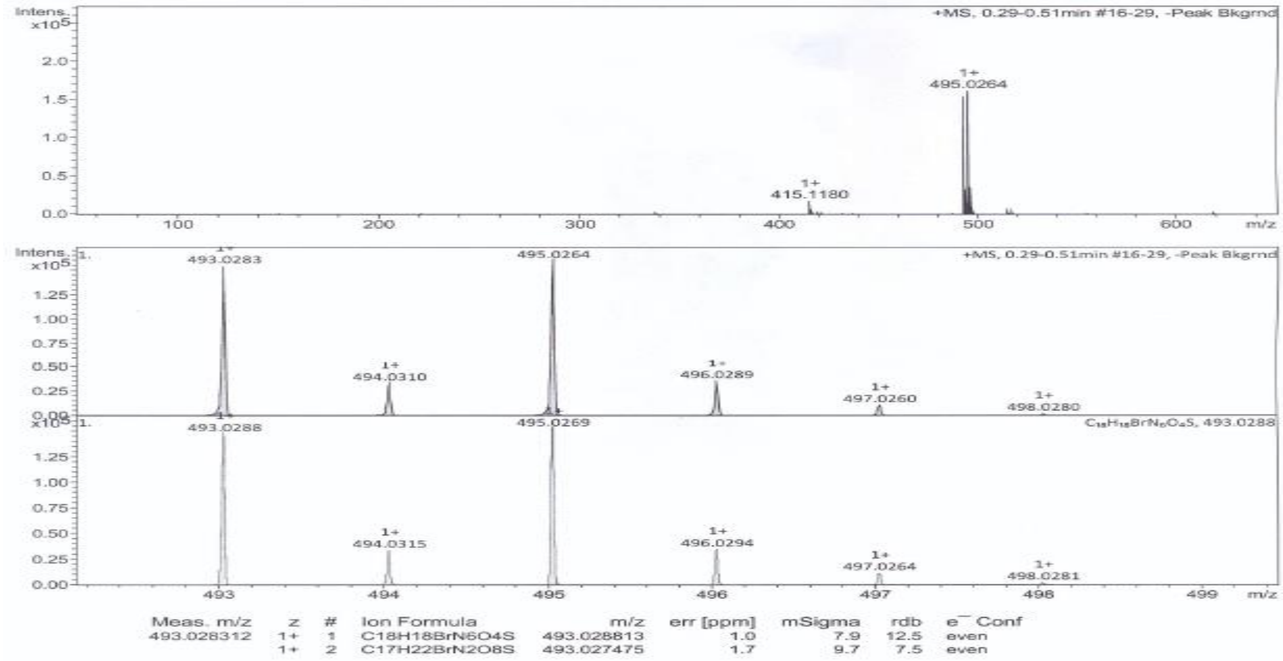

HRMS spectra of **6b**

2-(4-Chlorophenyl)-3-[(1,3-dimethylxanthin-7-yl)acethamido]thiazolidine-4-one (**6c**)

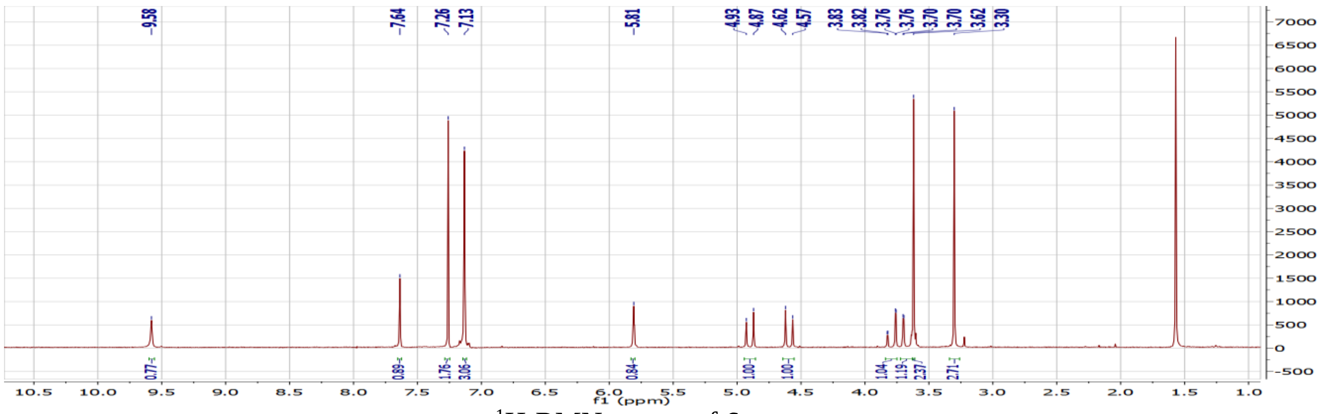

^{1}H-RMNspectra of **6c**

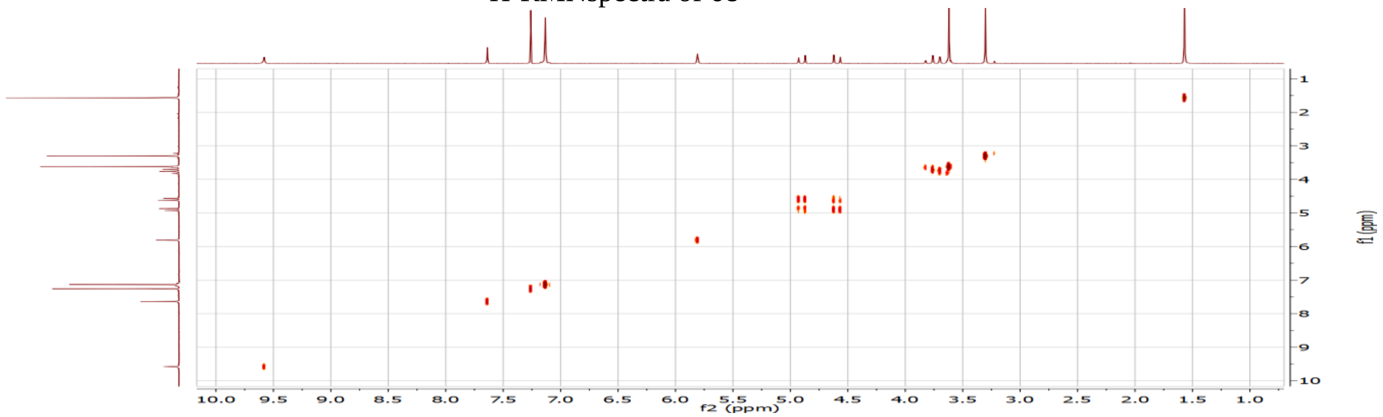

2D-COSYspectra of **6c**

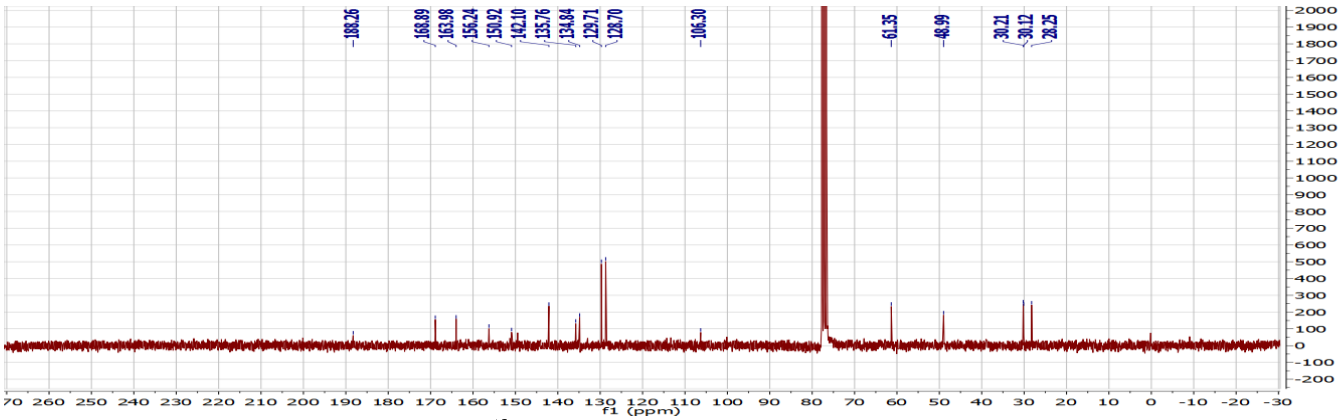

^{13}C-RMNspectra of **6c**

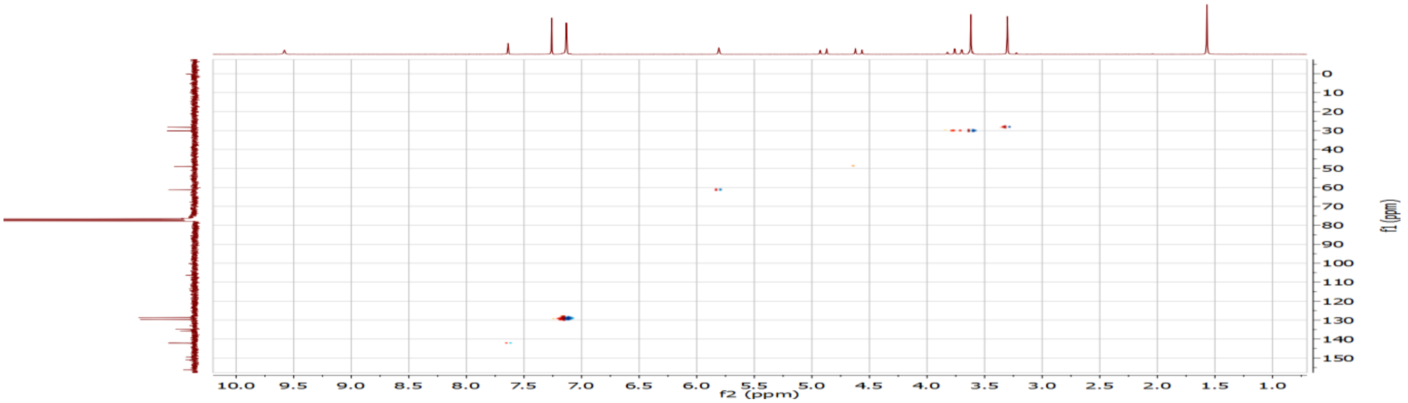

HSQC spectra of **6c**

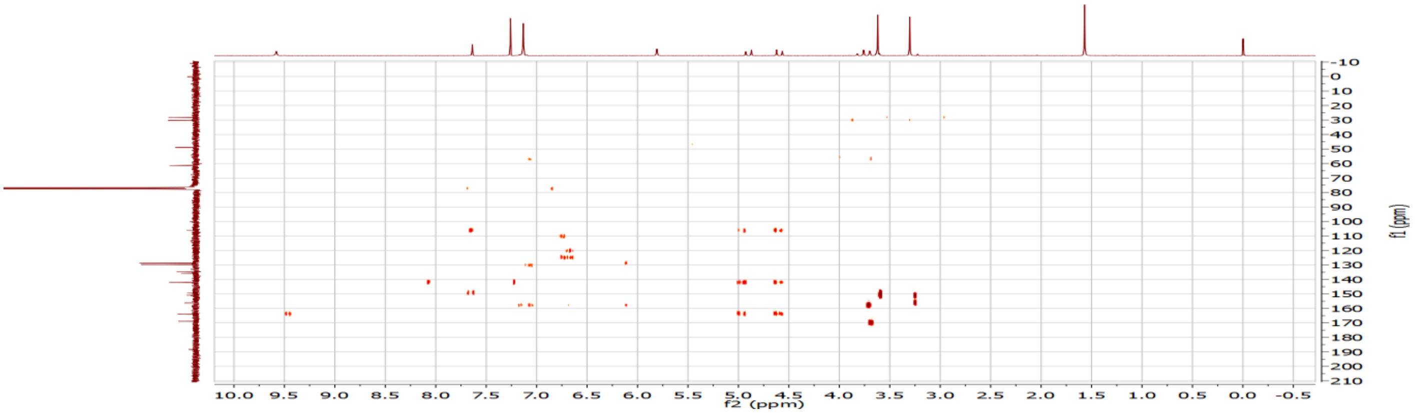

HMBC spectra of **6c**

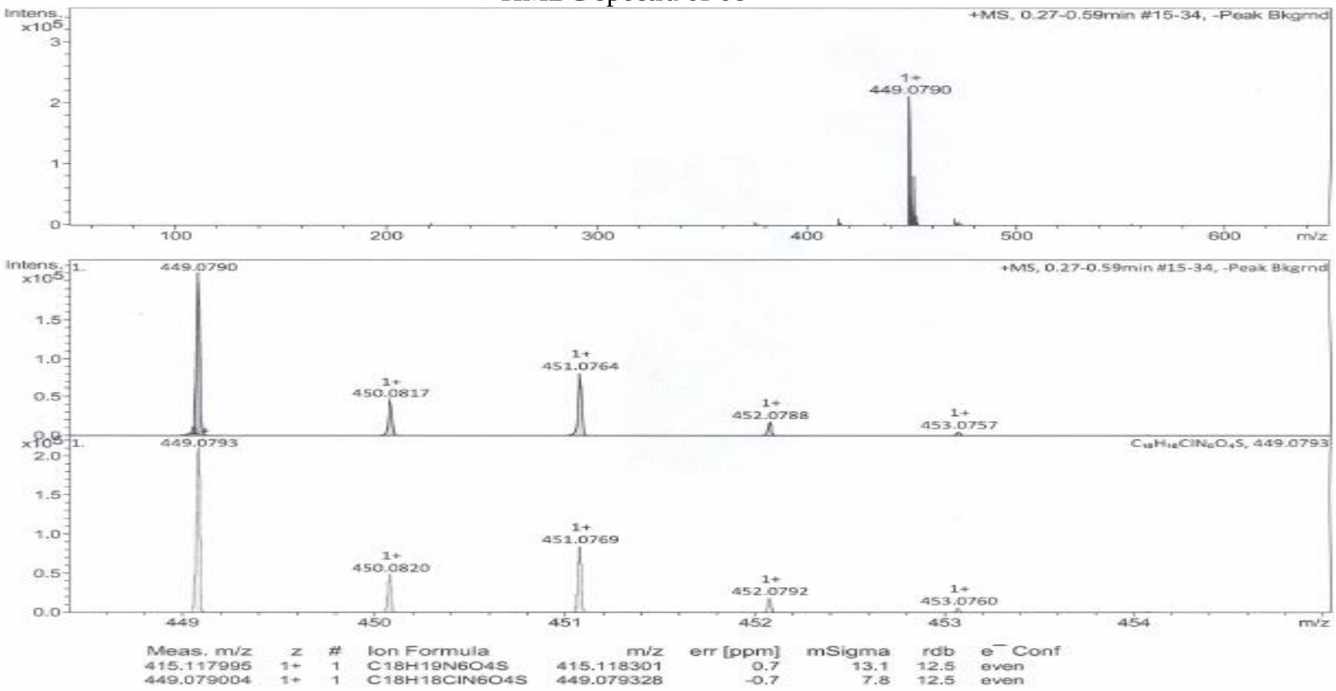

HRMS spectra of **6c**

2-(4-Fluorophenyl)-3-[(1,3-dimethylxanthin-7-yl)acethamido]thiazolidine-4-one (**6d**)

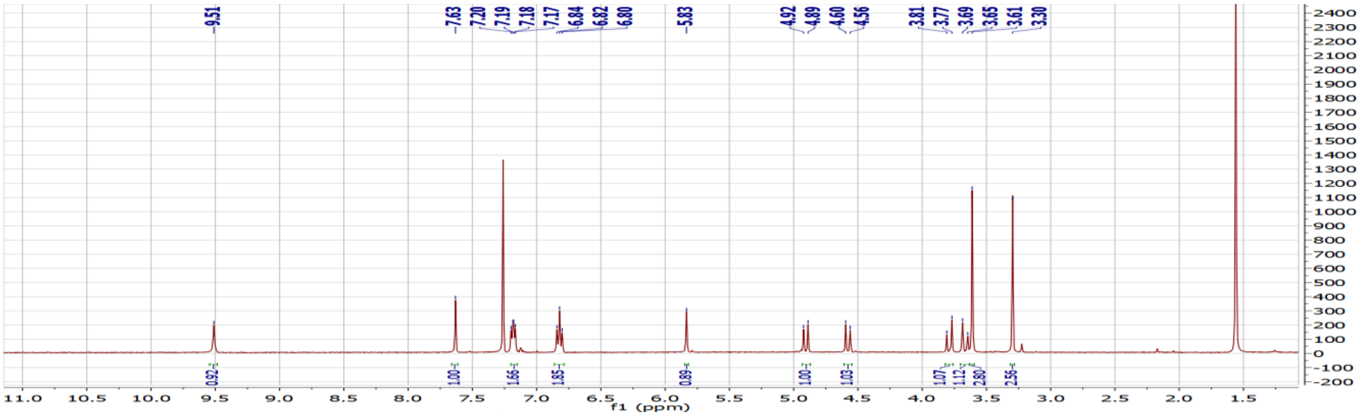

^{1}H-RMNspectra of **6d**

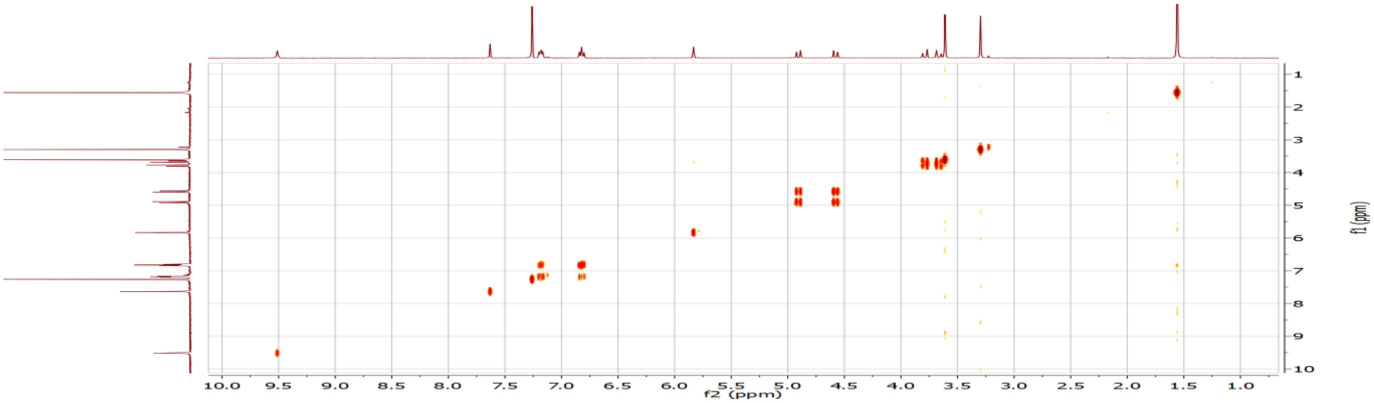

2D-COSYspectra of **6d**

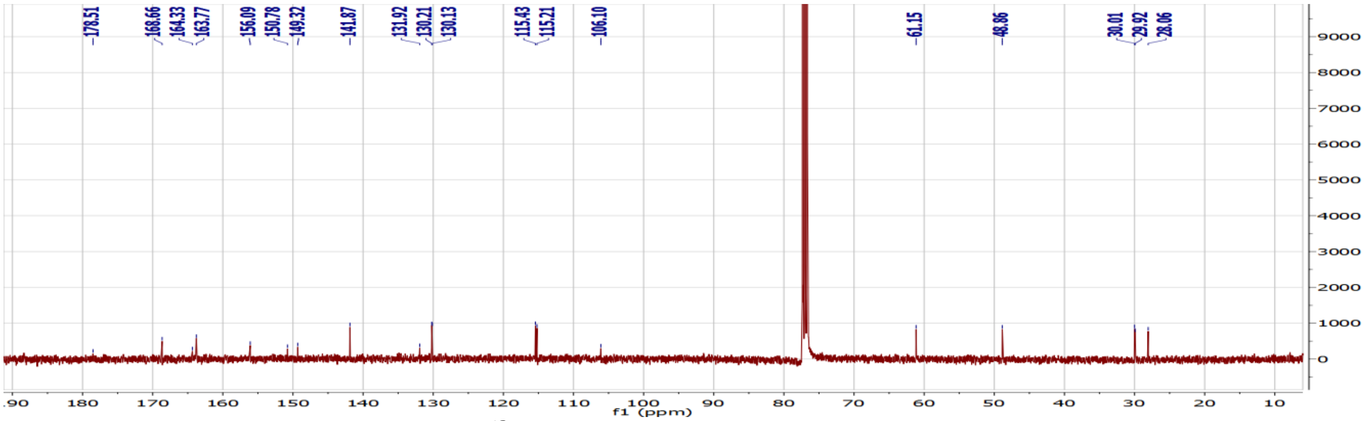

^{13}C-RMNspectra of **6d**

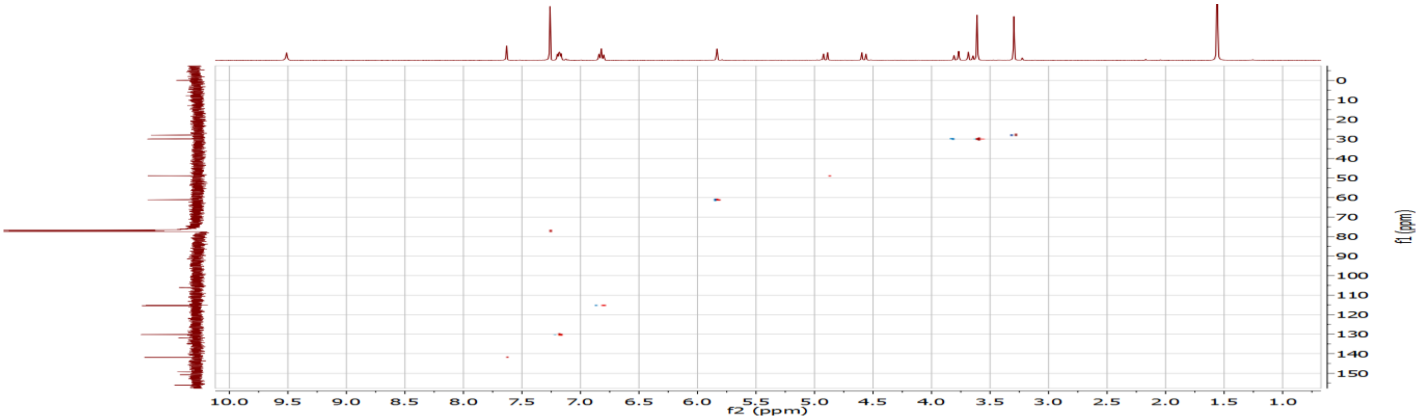

HSQC spectra of **6d**

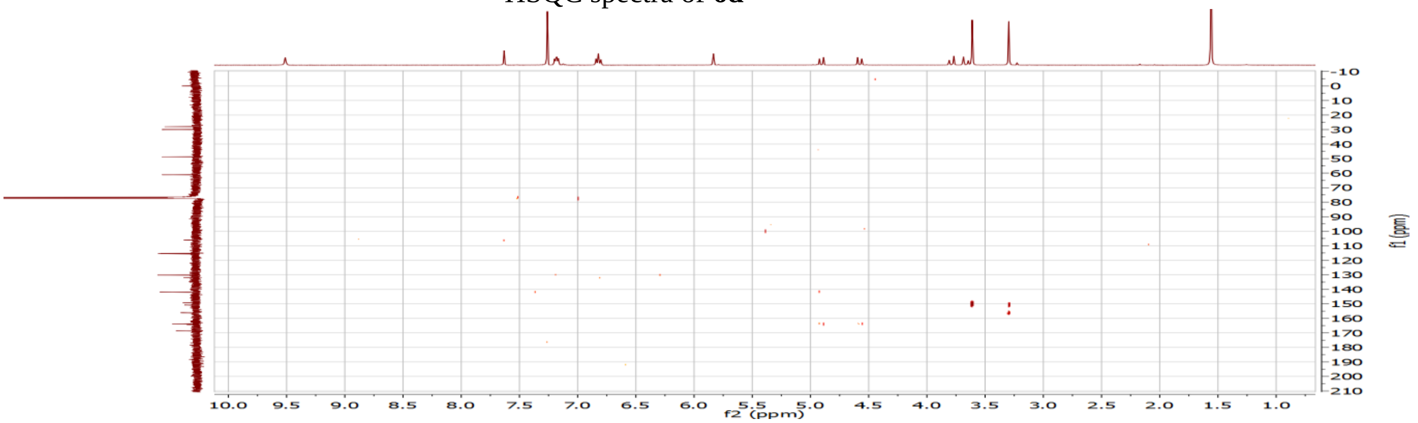

HMBC spectra of **6d**

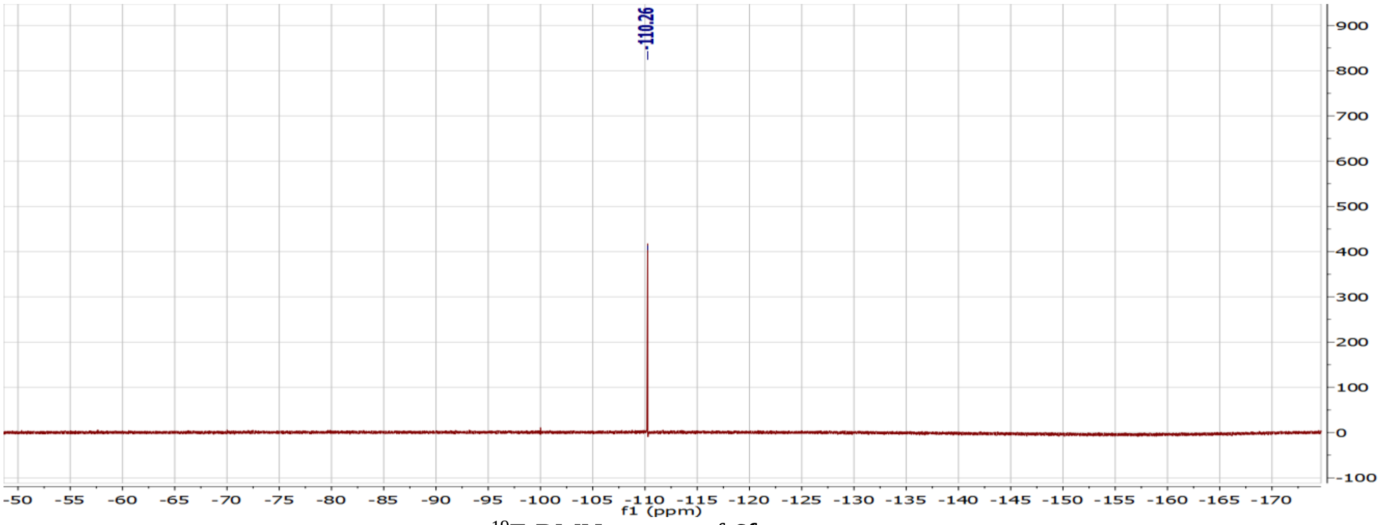

^{19}F-RMN spectra of **6f**

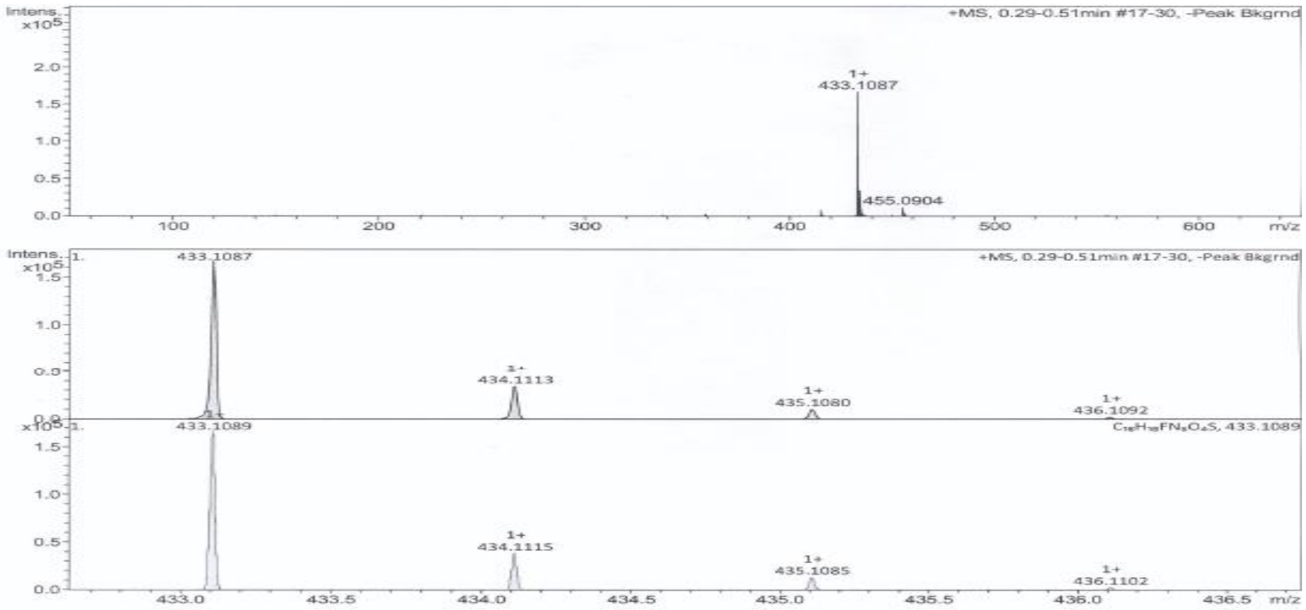

Meas. m/z	z	#	Ion Formula	m/z	err [ppm]	mSigma	rdb	e⁻ Conf
433.108662	1+	1	C18H18FN6O4S	433.108879	-0.5	14.0	12.5	even
455.090434	1+	1	C18H17FN6NaO4S	455.090823	-0.9	4.2	12.5	even
	1+	2	C21H16N6NaO3S	455.089680	-1.7	15.3	16.5	even

HRMS spectra of **6d**

2-(2-Methoxyphenyl)-3-[(1,3-dimethylxanthin-7-yl)acethamido]thiazolidine-4-one (**6e**)

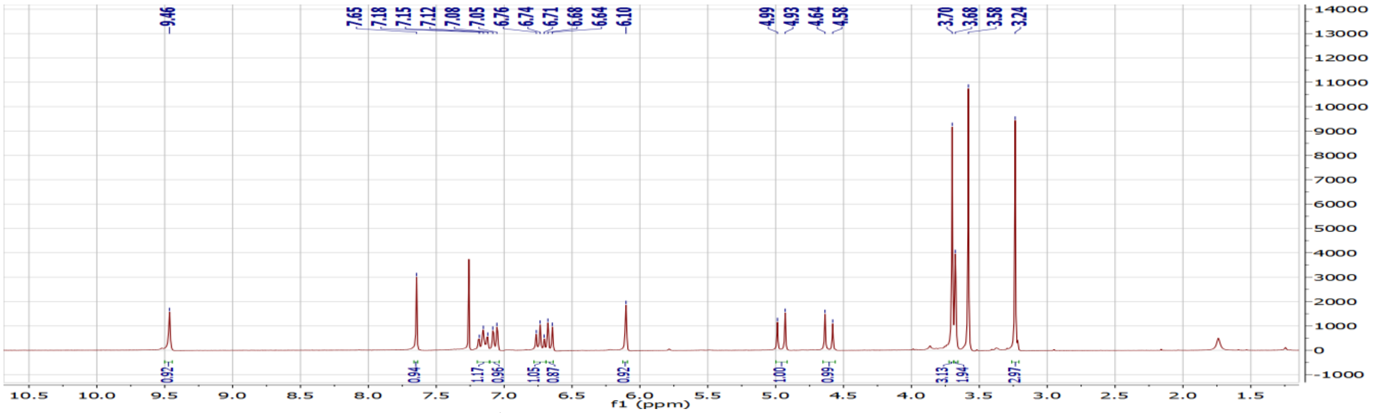

^{1}H-RMNspectra of **6e**

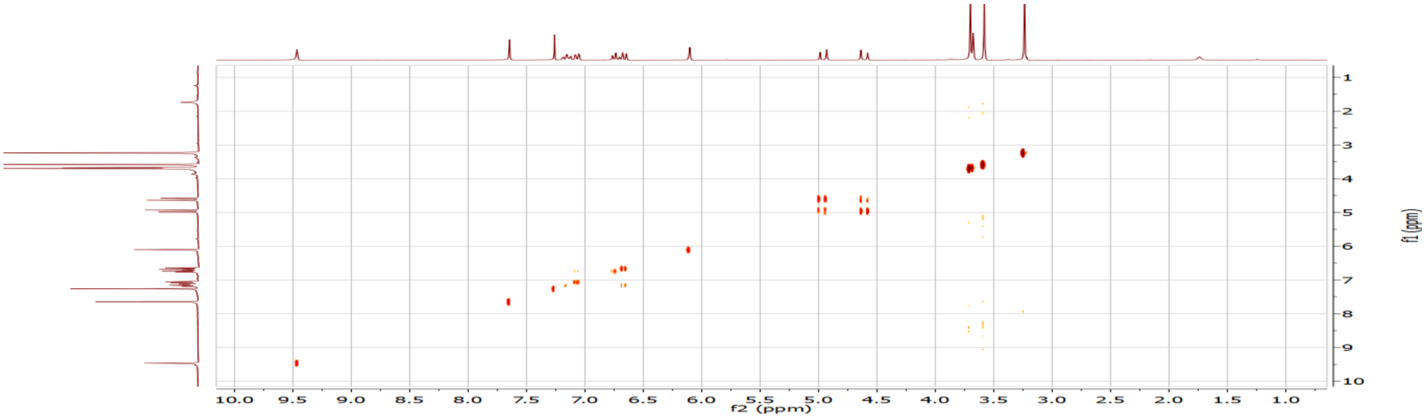

2D-COSYspectra of **6e**

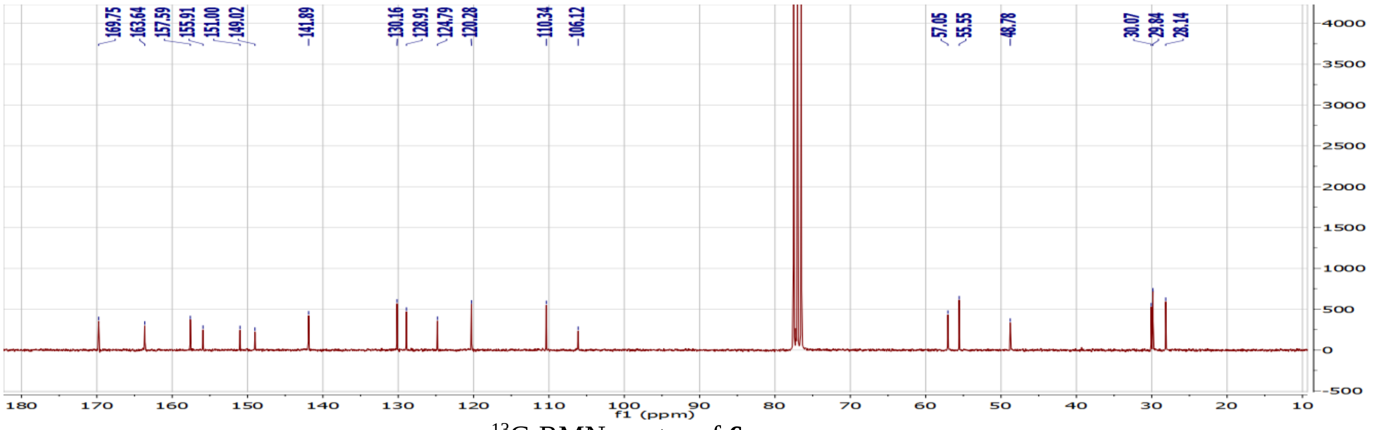

^{13}C-RMNspectra of **6e**

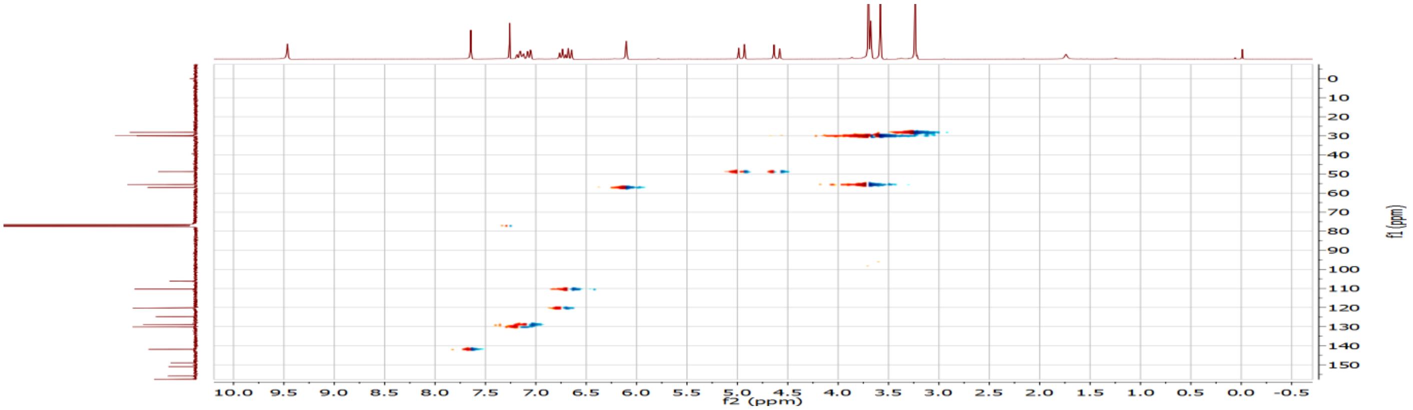

HSQC spectra of **6e**

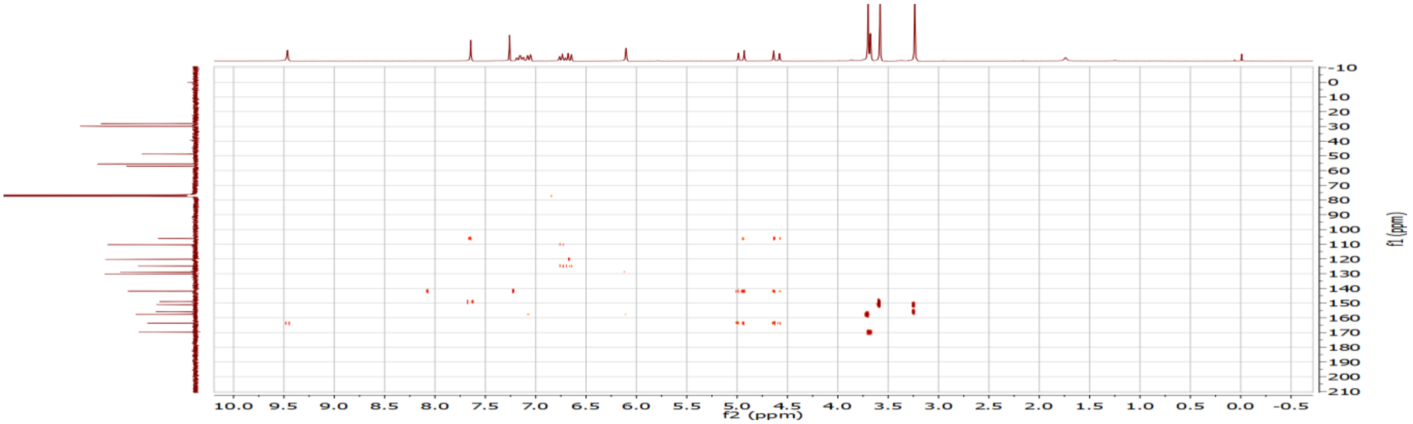

HMBC spectra of **6e**

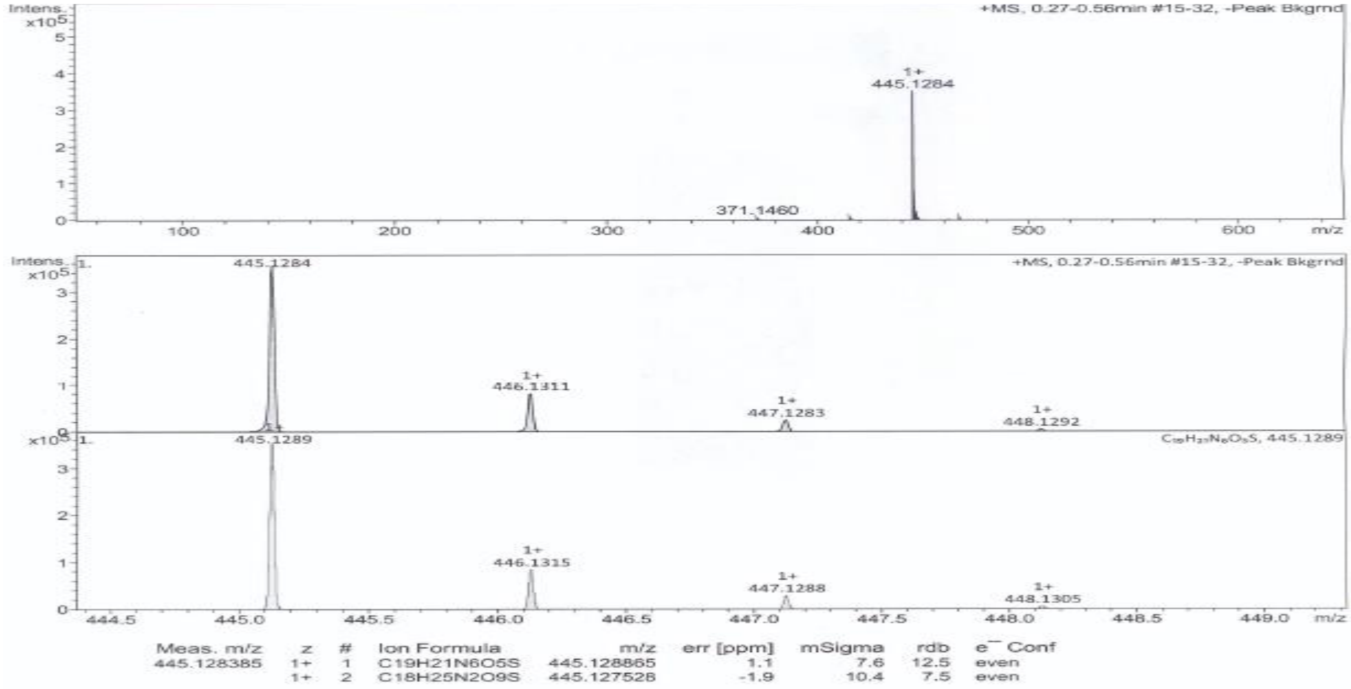

HRMS spectra of **6e**

2-(4-Methoxyphenyl)-3-[(1,3-dimethylxanthin-7-yl)acethamido]thiazolidine-4-one (**6f**)

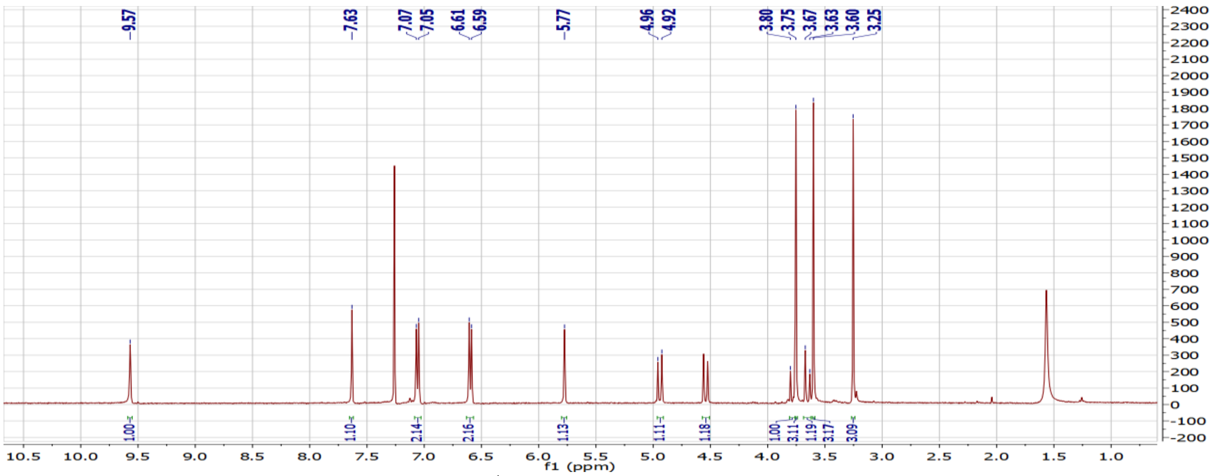

^{1}H-RMN spectra of **6f**

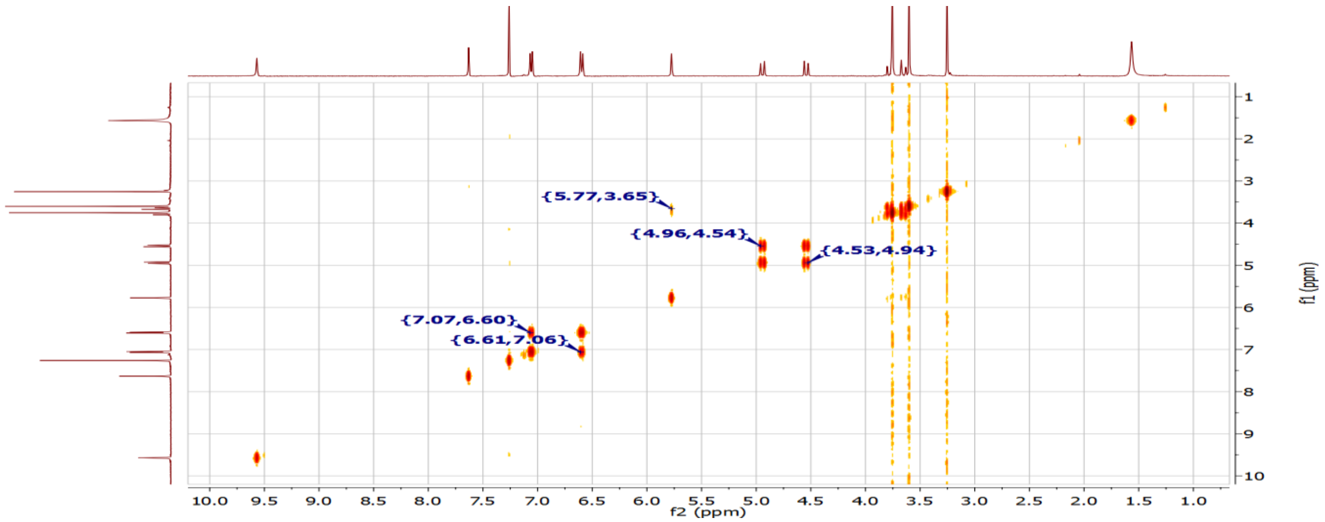

2D-COSY spectra of **6f**

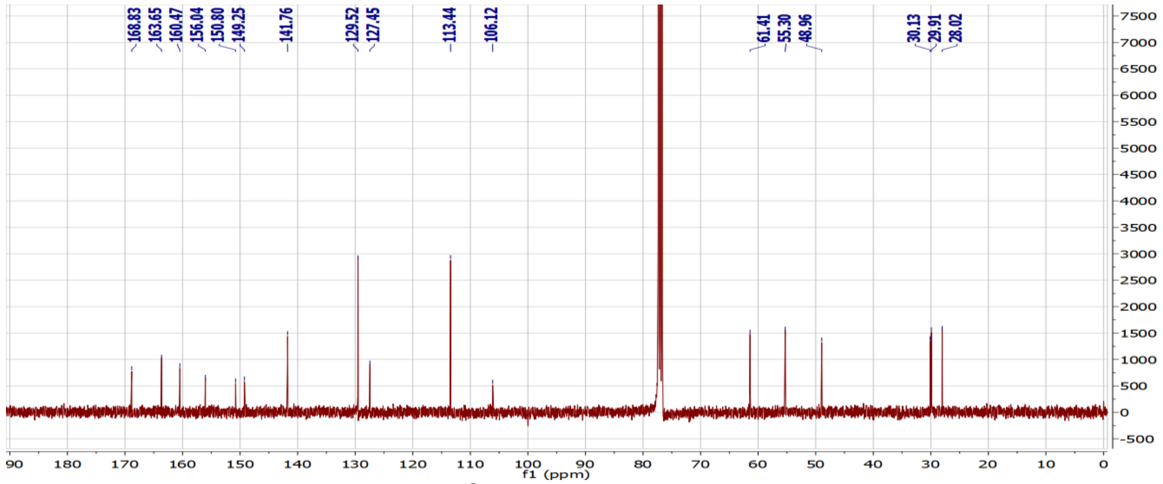

^{13}C-RMN spectra of **6f**

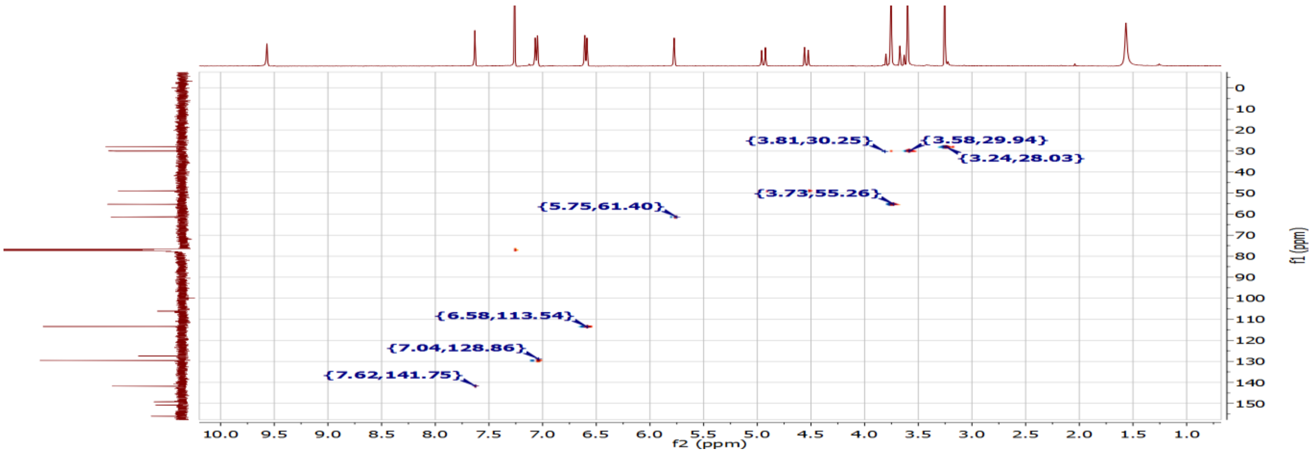

HSQC spectra of **6f**

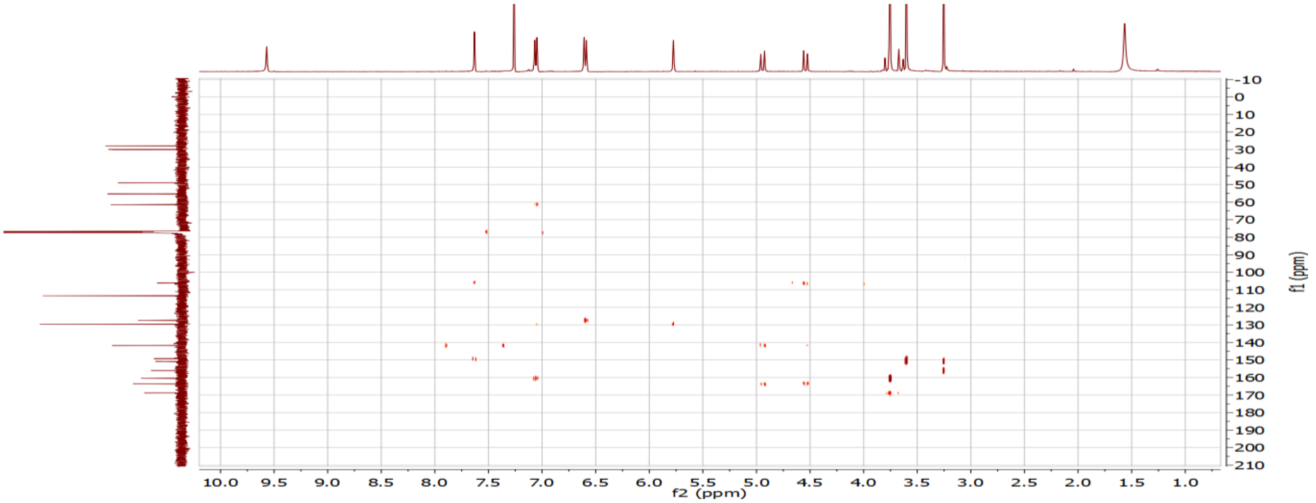

HMBC spectra of **6f**

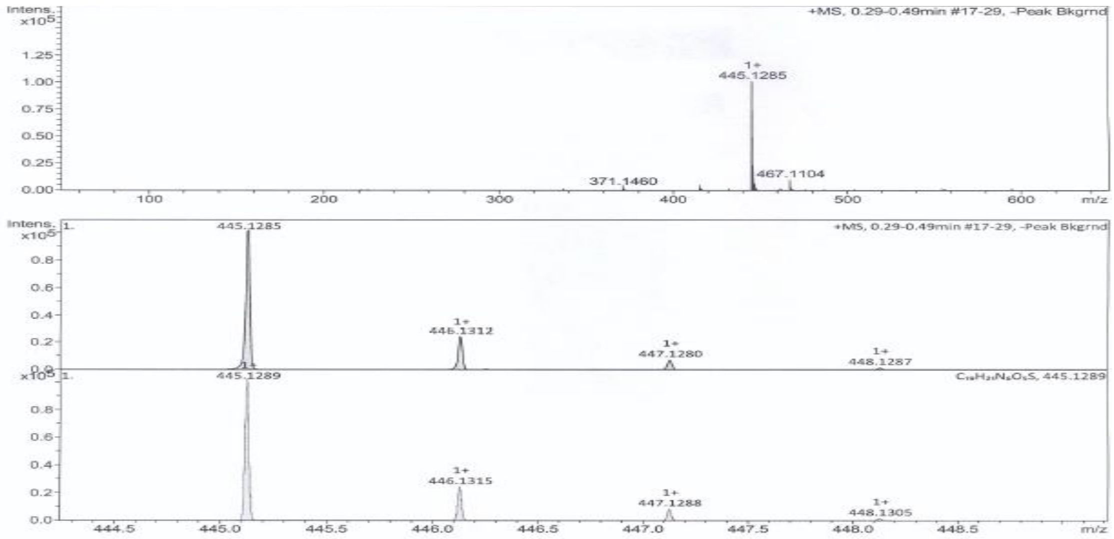

Meas. m/z	z	#	Ion Formula	m/z	err [ppm]	mSigma	rdb	e⁻ Conf
445.128533	1+	1	C19H21N6O5S	445.128865	0.7	7.8	12.5	even
467.110416	1+	1	C19H20N6NaO5S	467.110809	-0.8	4.3	12.5	even

HRMS spectra forof **6f**

<u>*2-(3-Methoxyphenyl)-3-[(1,3-dimethylxanthin-7-yl)acethamido]thiazolidine-4-one* (**6g**)</u>

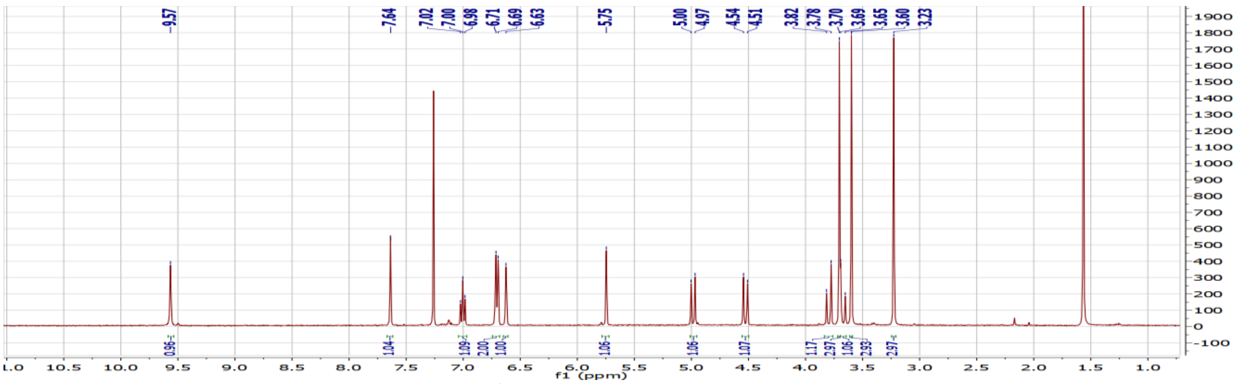

^{1}H-RMNspectra of **6g**

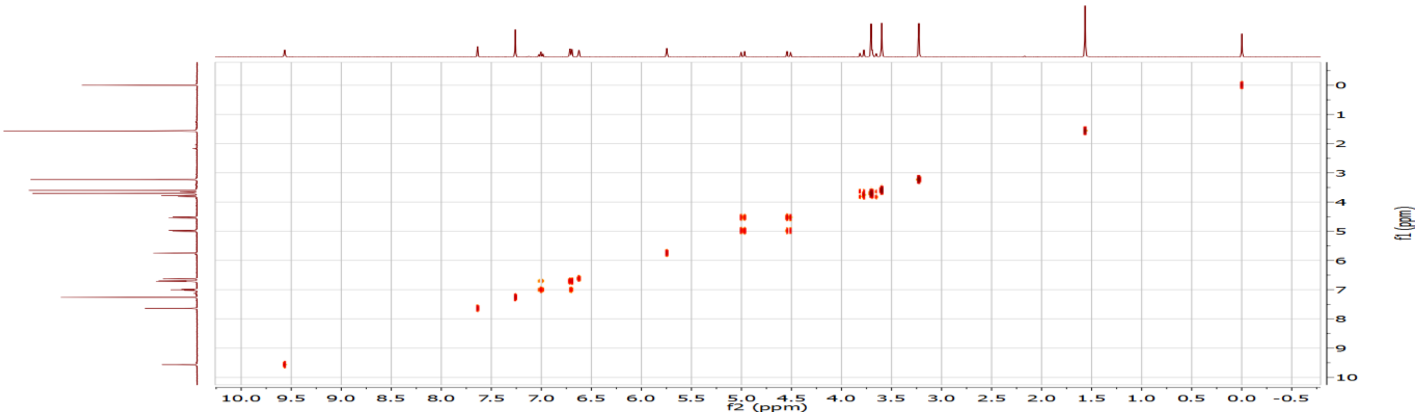

2D-COSYspectra of **6g**

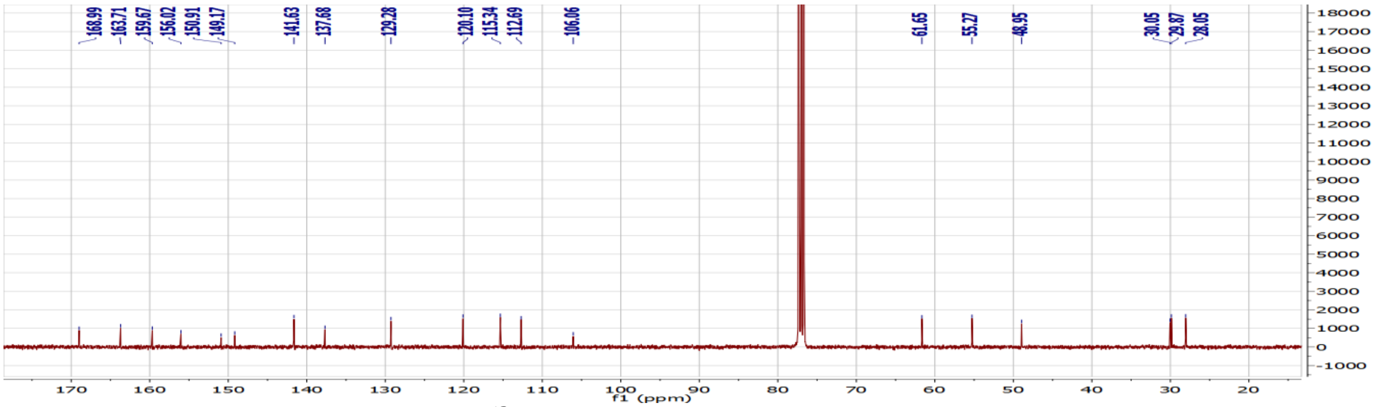

^{13}C-RMNspectra of **6g**

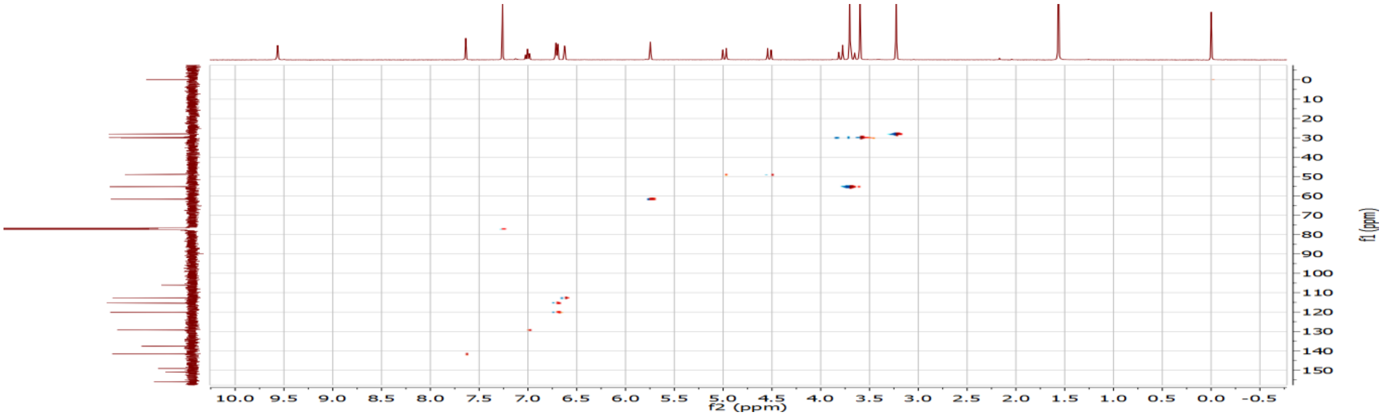

HSQC spectra of **6g**

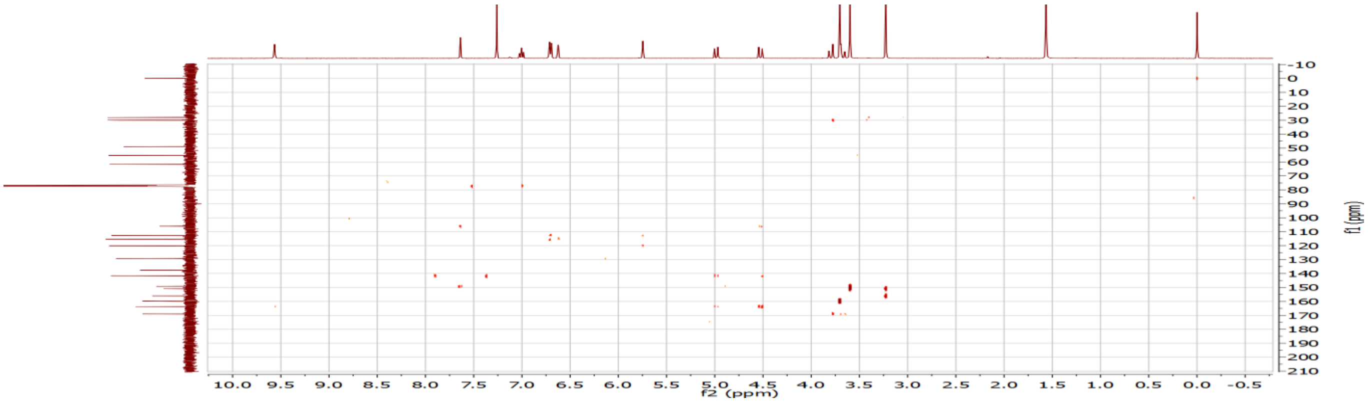

HMBC spectra of **6g**

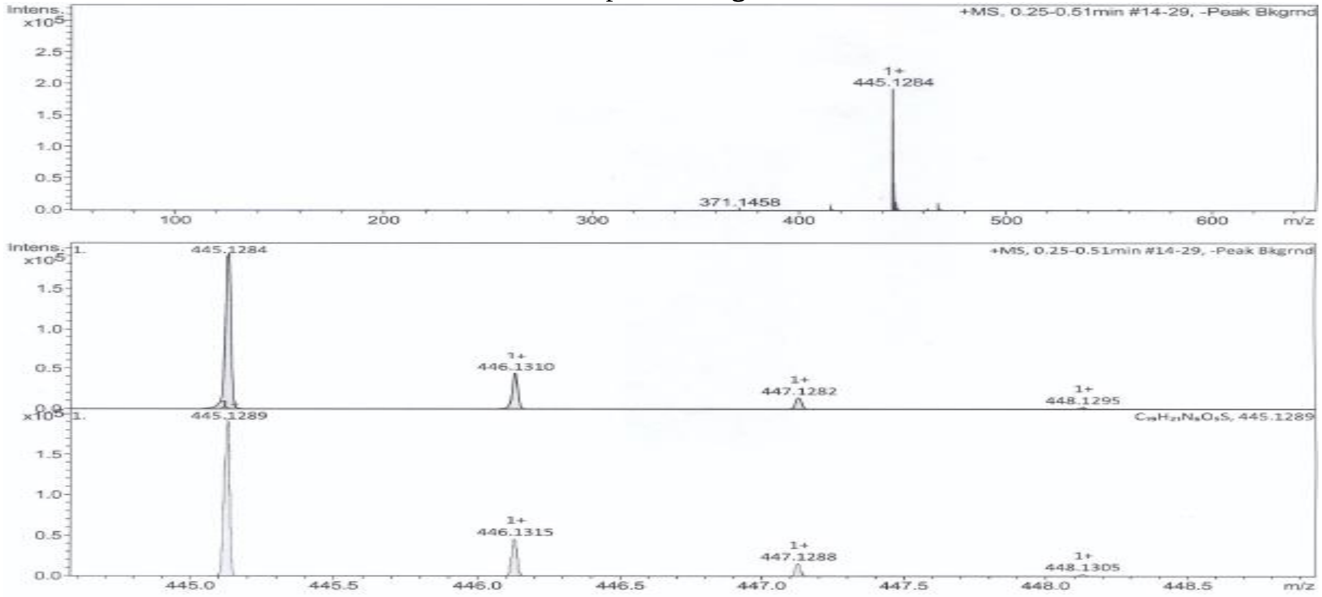

Meas. m/z	z	#	Ion Formula	m/z	err [ppm]	mSigma	rdb	e⁻ Conf
445.128366	1+	1	C19H21N6O5S	445.128865	-1.1	7.5	12.5	even
	1+	2	C18H25N2O9S	445.127528	-1.9	10.0	7.5	even
467.110205	1+	1	C18H24N2NaO9S	467.109472	1.6	12.2	7.5	even
	1+	2	C19H20N6NaO5S	467.110809	1.3	16.0	12.5	even

HRMS spectra of **6g**

2-(2,3-Dimethoxyphenyl)-3-[(1,3-dimethylxanthin-7-yl)acethamido]thiazolidine-4-one (**6h**)

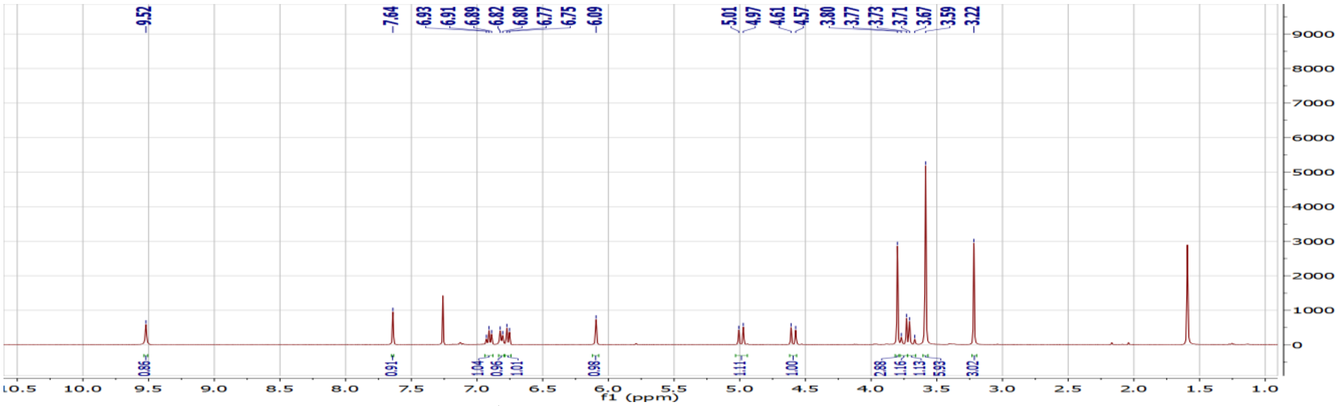

^{1}H-RMNspectra of **6h**

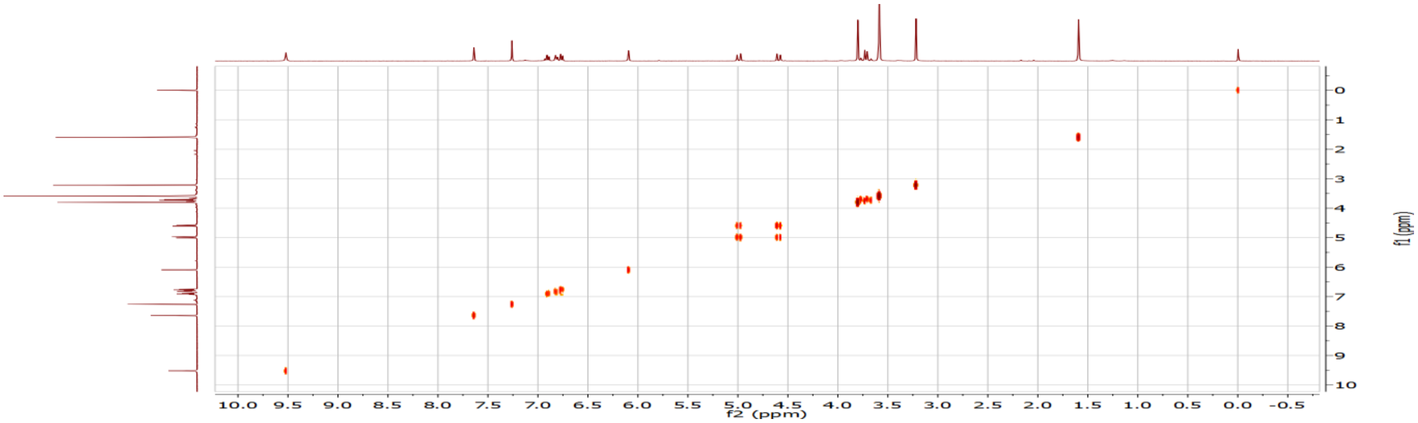

2D-COSYspectra of **6h**

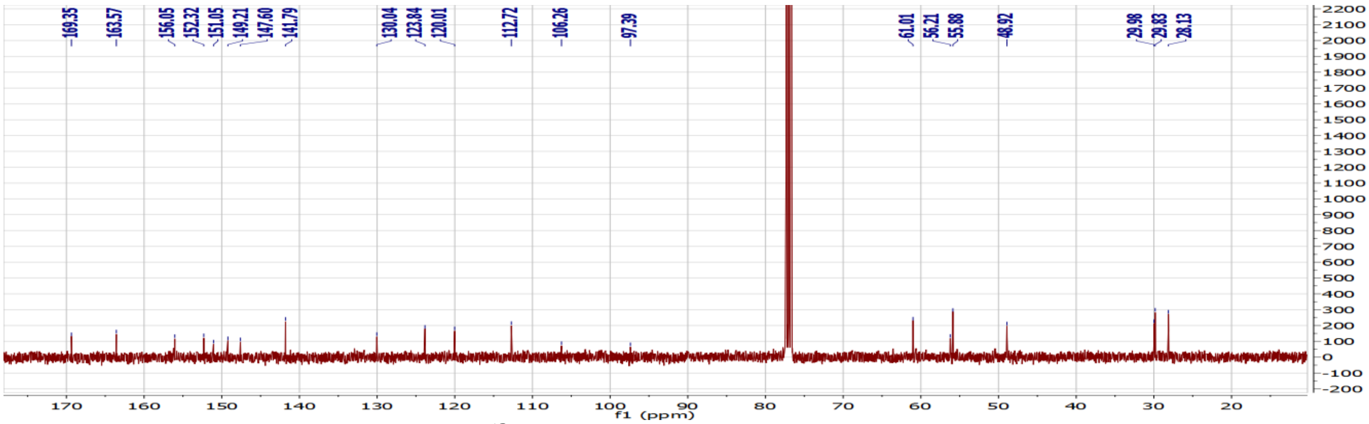

^{13}C-RMNspectra of **6h**

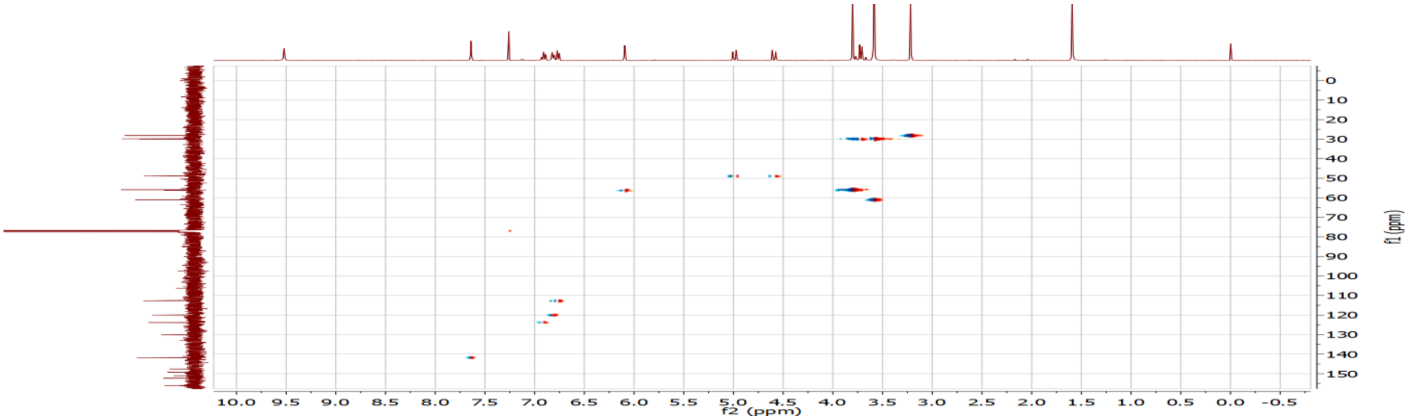

HSQC spectra of **6h**

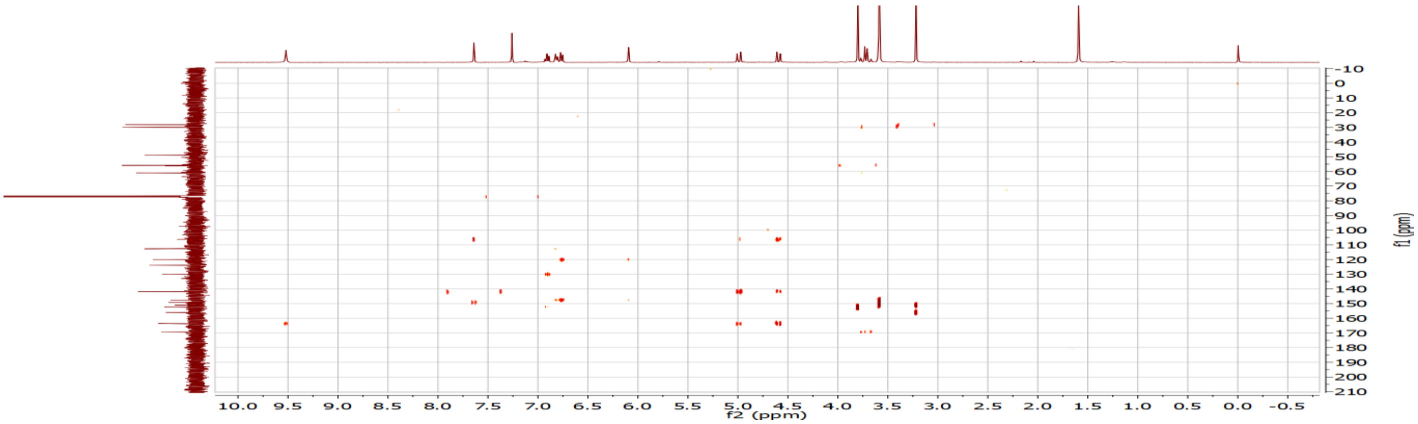

HMBC spectra of **6h**

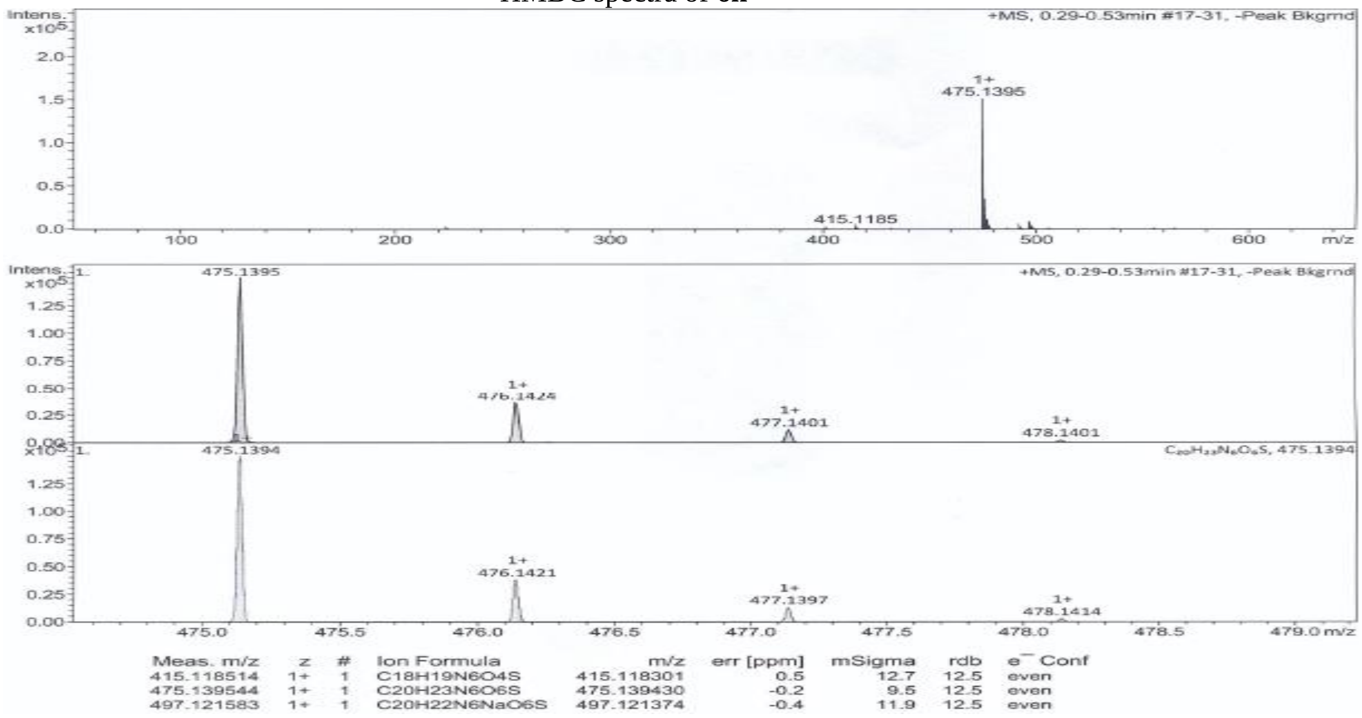

HRMS spectra of **6h**

<u>*2-(2,4-Dimethoxyphenyl)-3-[(1,3-dimethylxanthin-7-yl)acethamido]thiazolidine-4-one* (**6i**)</u>

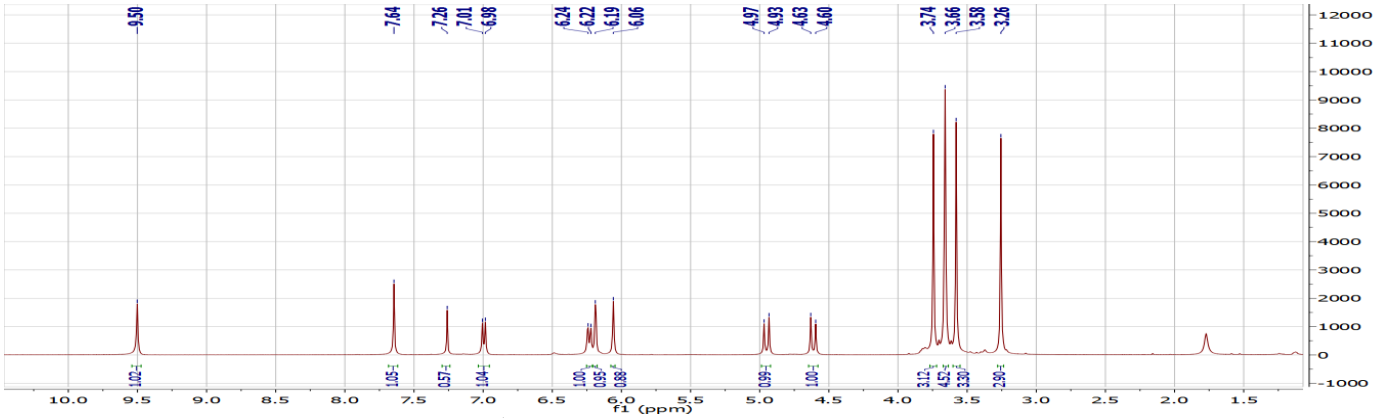

^{1}H-RMNspectra of **6i**

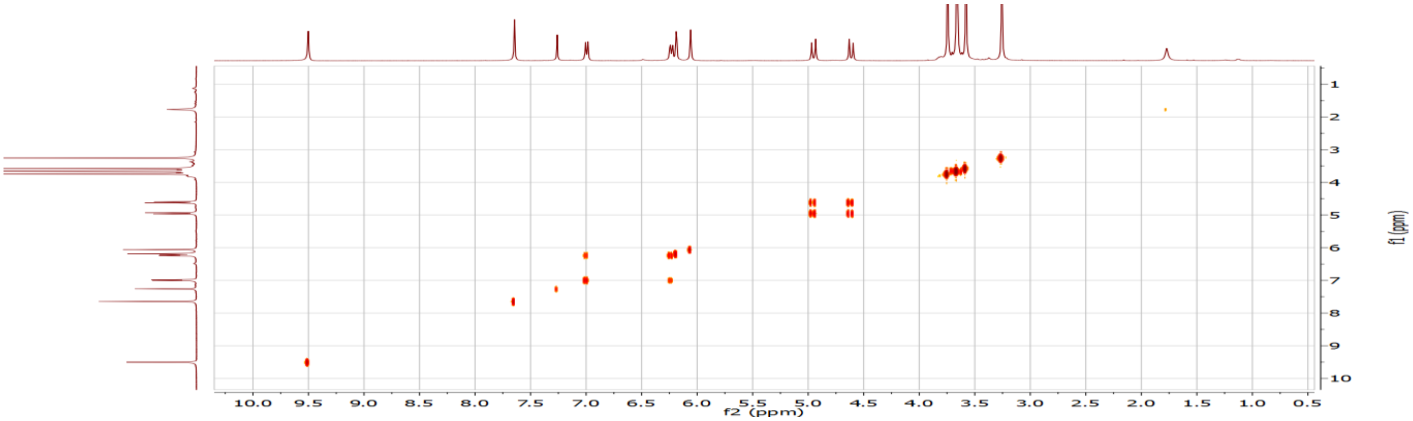

2D-COSYspectra of **6i**

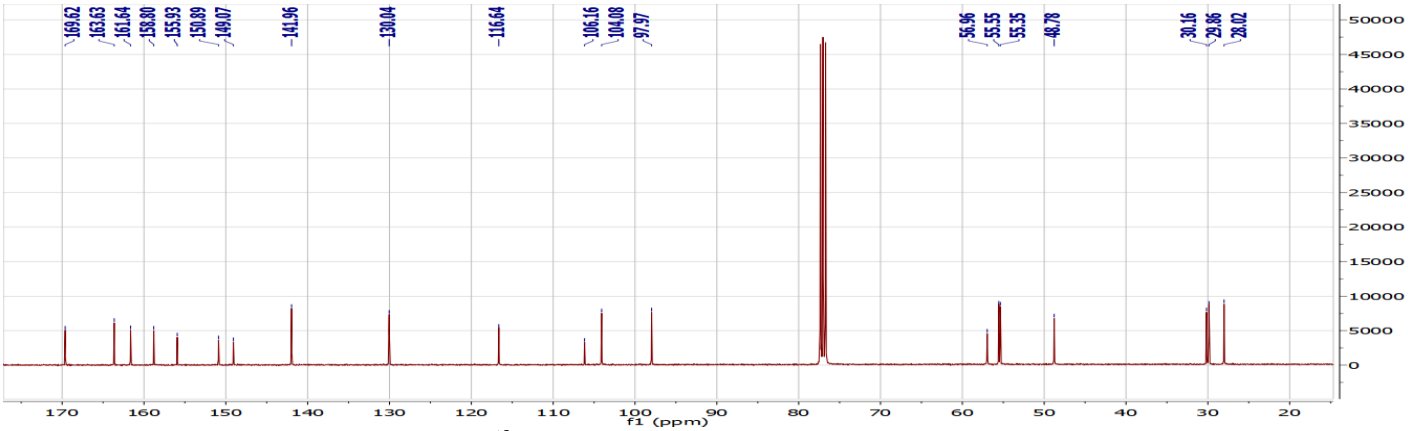

^{13}C-RMNspectra of **6i**

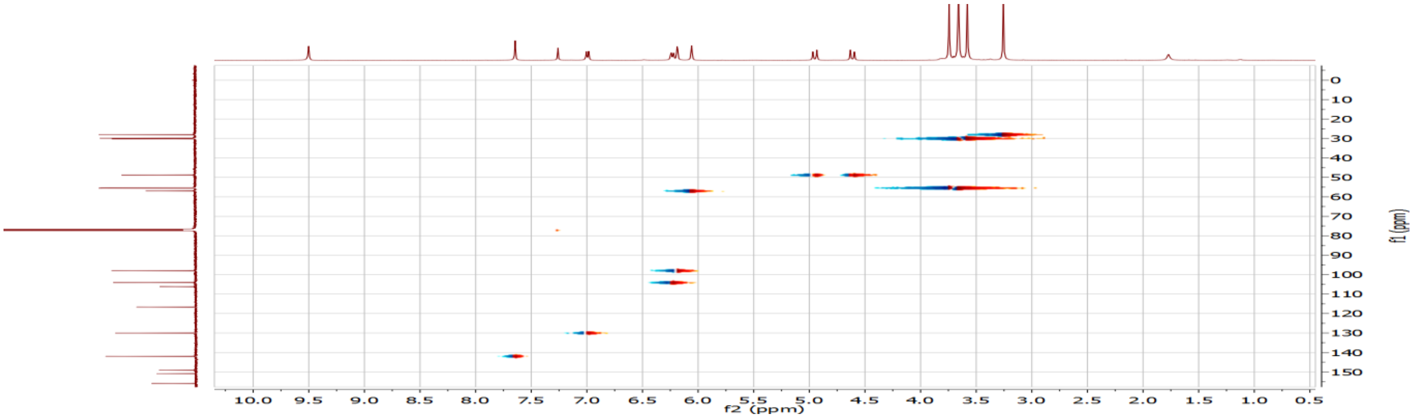

HSQC spectra of **6i**

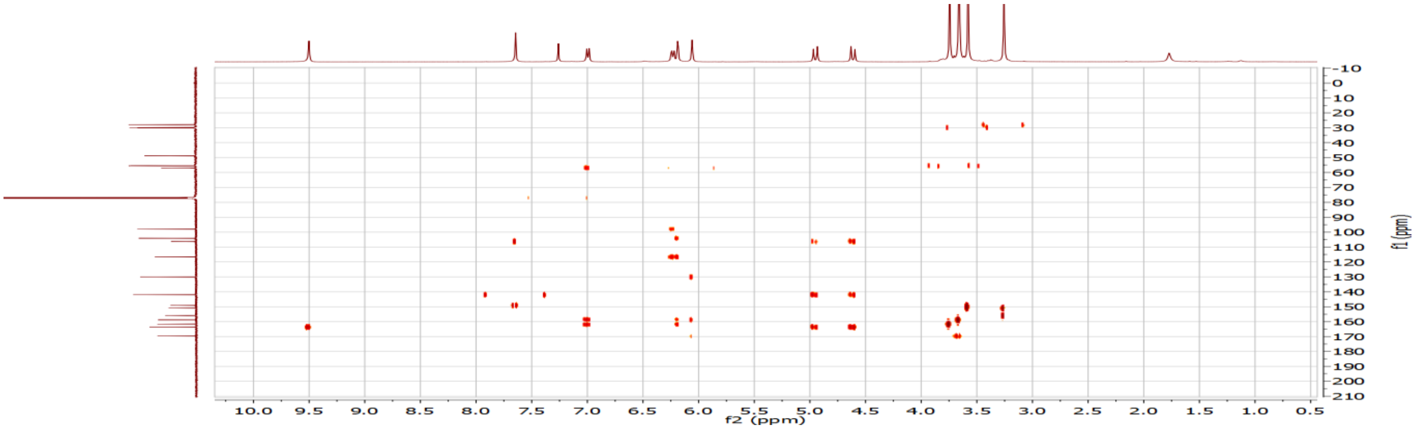

HMBC spectra of **6i**

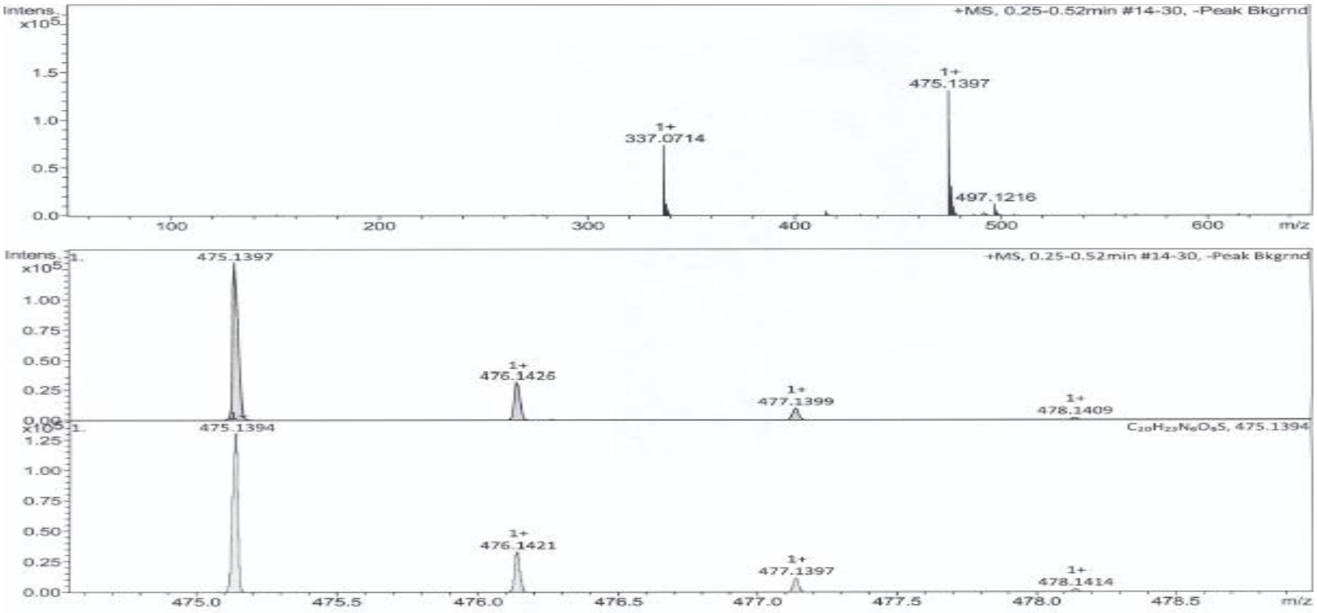

Meas. m/z	z	#	Ion Formula	m/z	err [ppm]	mSigma	rdb	e⁻ Conf
337.071445	1+	1	C12H13N6O4S	337.071350	-0.3	6.0	9.5	even
475.139685	1+	1	C20H23N6O6S	475.139430	0.5	12.0	12.5	even
497.121569	1+	1	C20H22N6NaO6S	497.121374	-0.4	7.5	12.5	even

HRMSspectra of **6i**

2-(3,5-Dimethoxyphenyl)-3-[(1,3-dimethylxanthin-7-yl)acethamido]thiazolidine-4-one (**6j**)

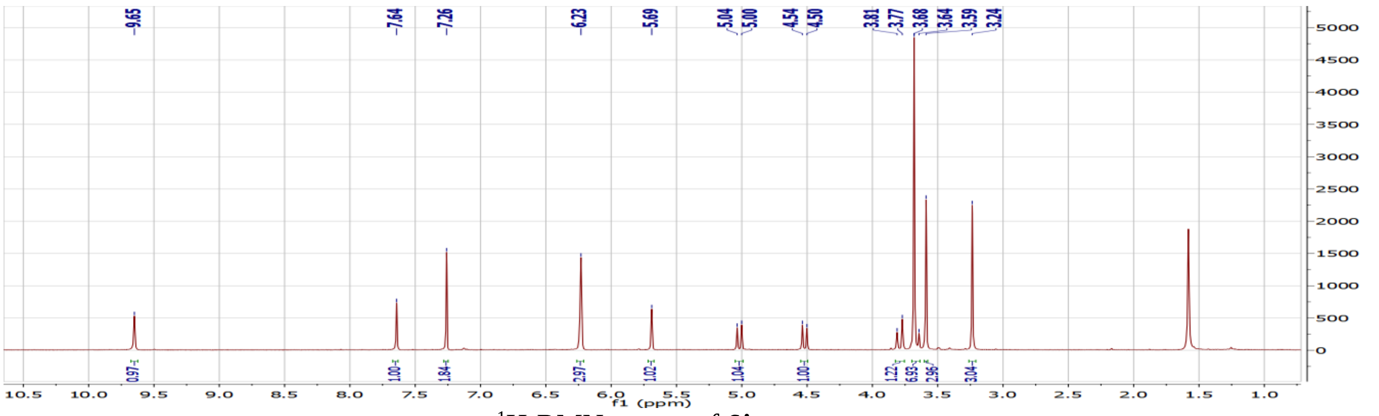

^{1}H-RMNspectra of **6j**

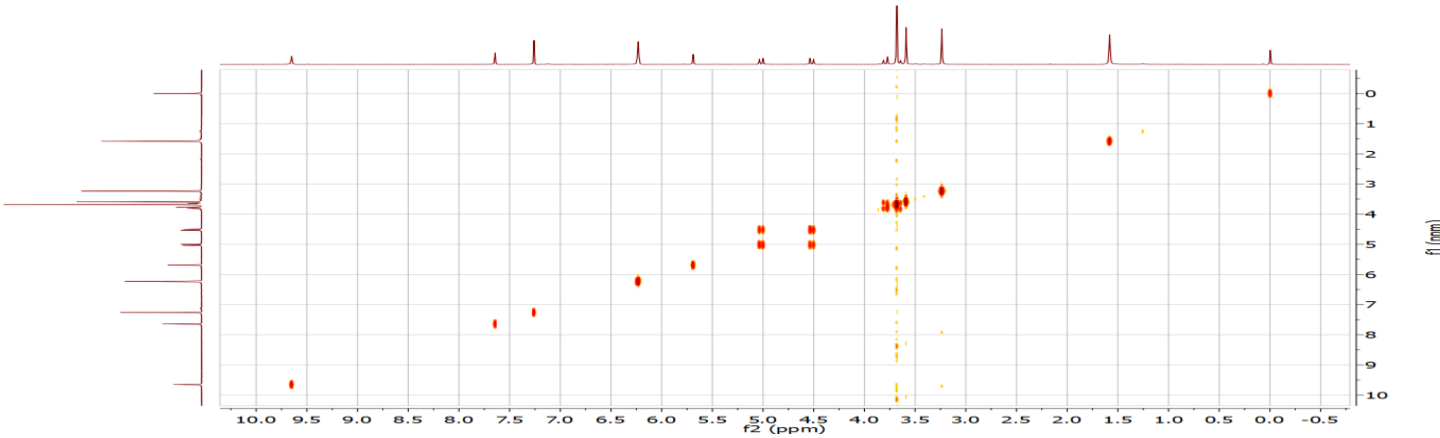

2D-COSYspectra of **6j**

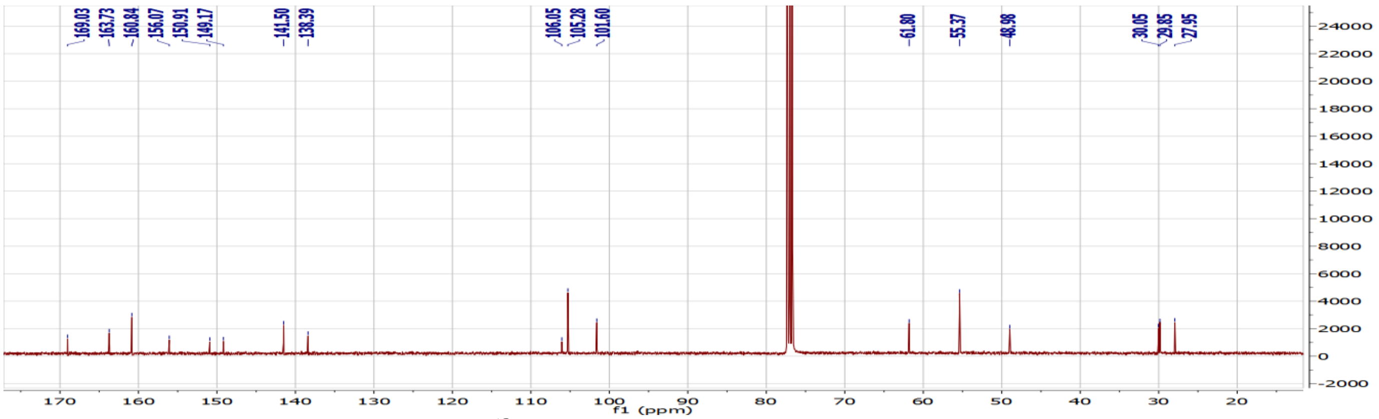

^{13}C-RMNspectra of **6j**

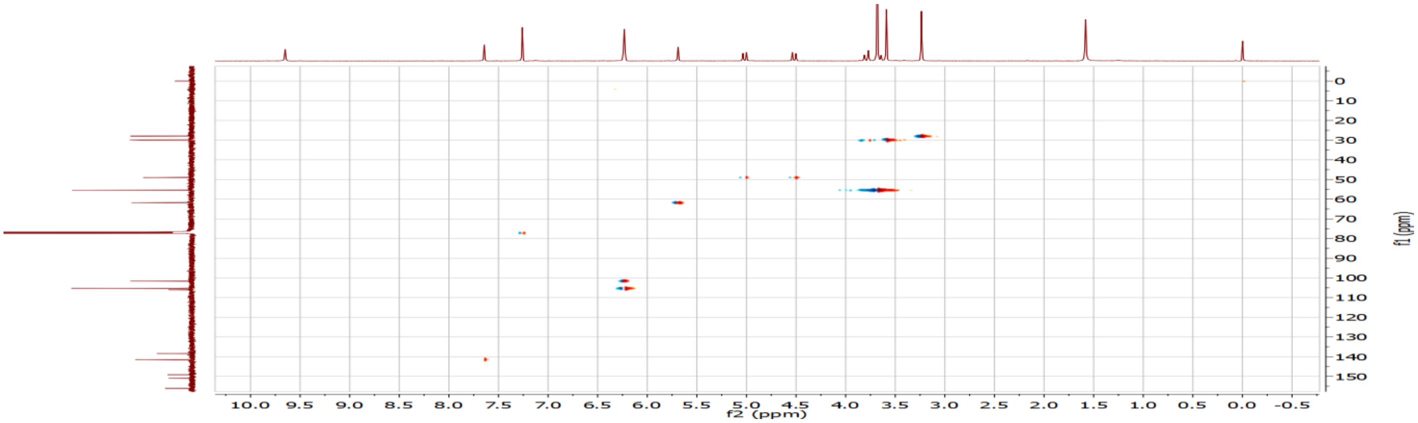

HSQC spectra of **6j**

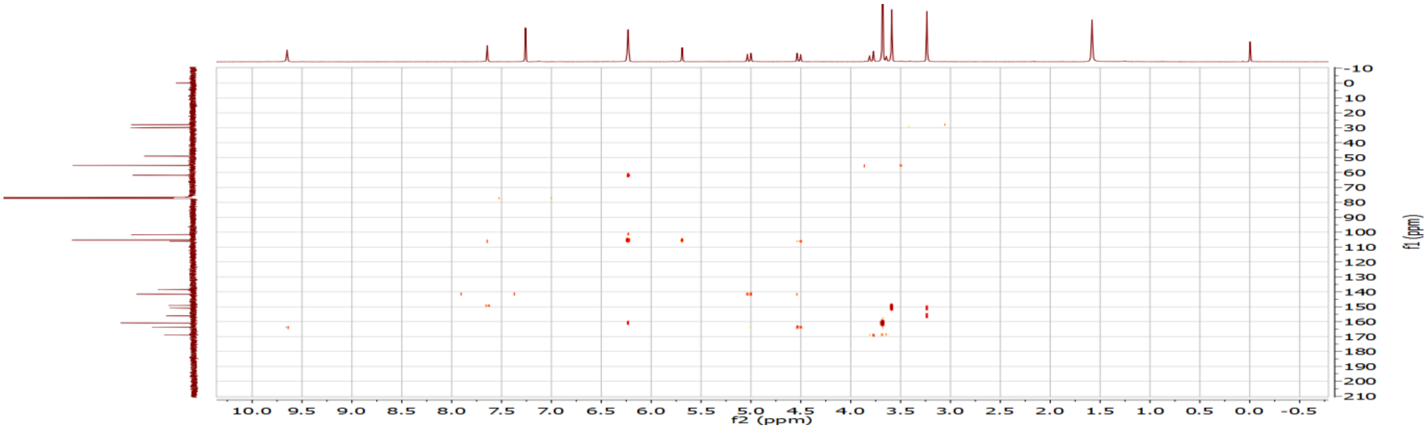

HMBC spectra of **6j**

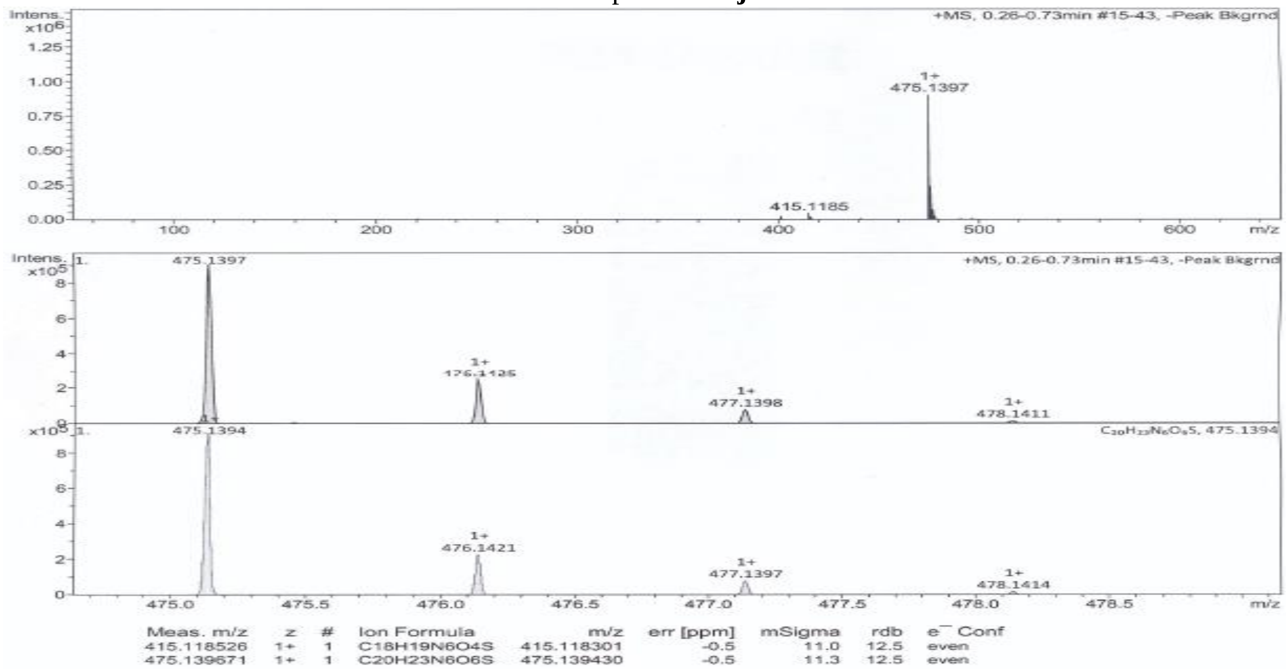

HRMS spectra of **6j**

<u>*2-(4-Methylphenyl)-3-[(1,3-dimethylxanthin-7-yl)acethamido]thiazolidine-4-one* (**6k**)</u>

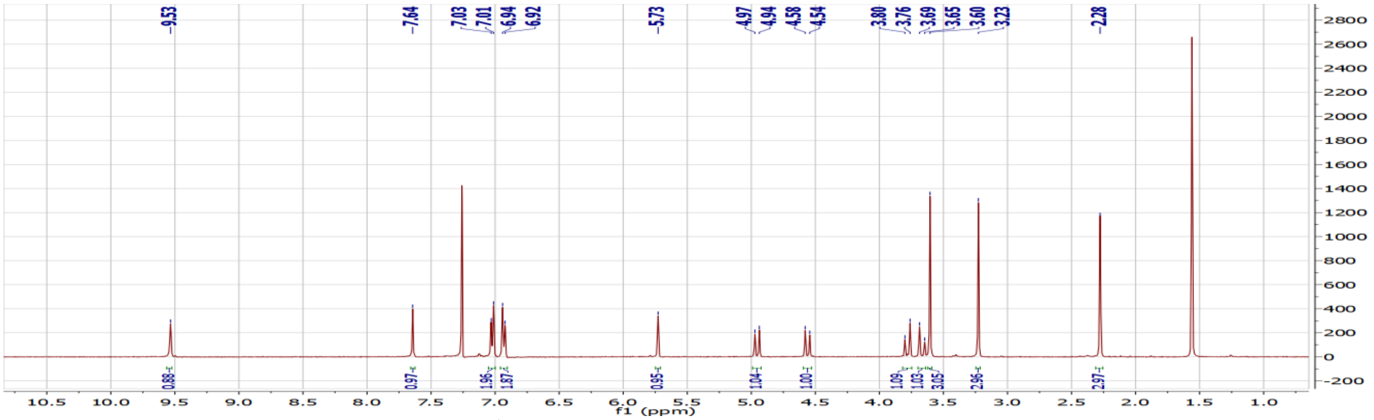

^{1}H-RMNspectra of **6k**

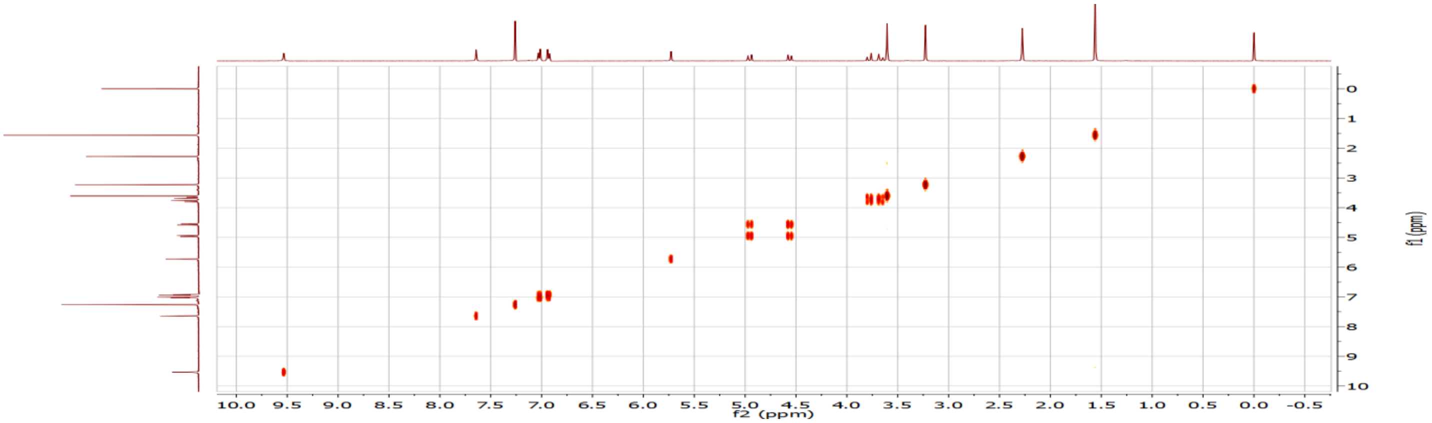

2D-COSYspectra of **6k**

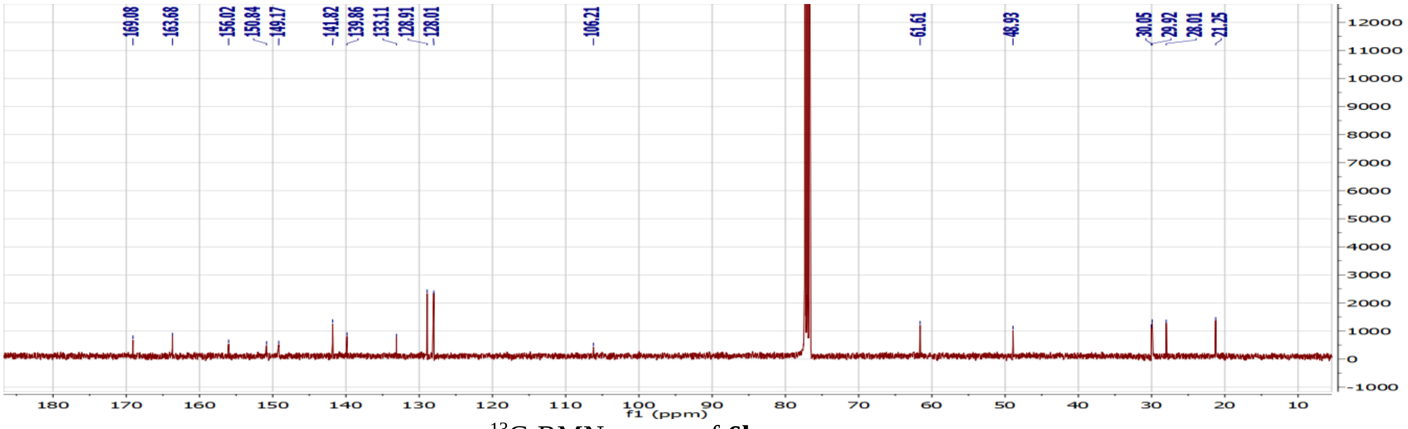

^{13}C-RMNspectra of **6k**

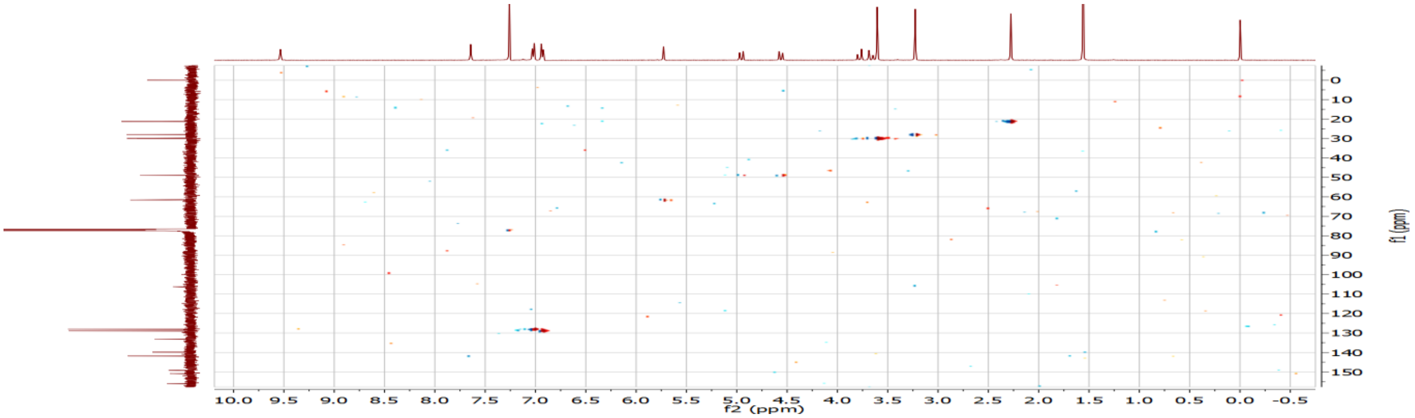

HSQC spectra of **6k**

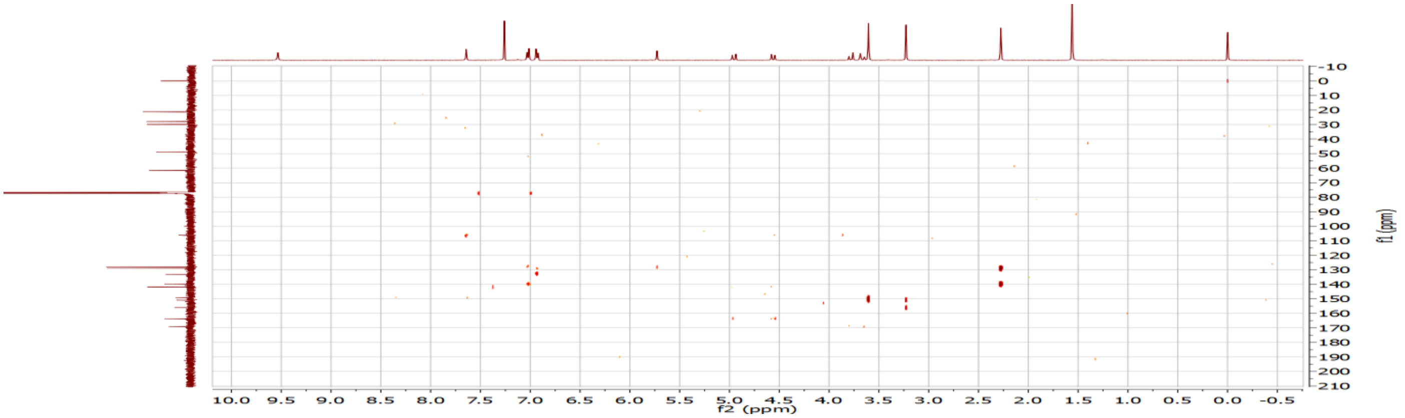

HMBC spectra of **6k**

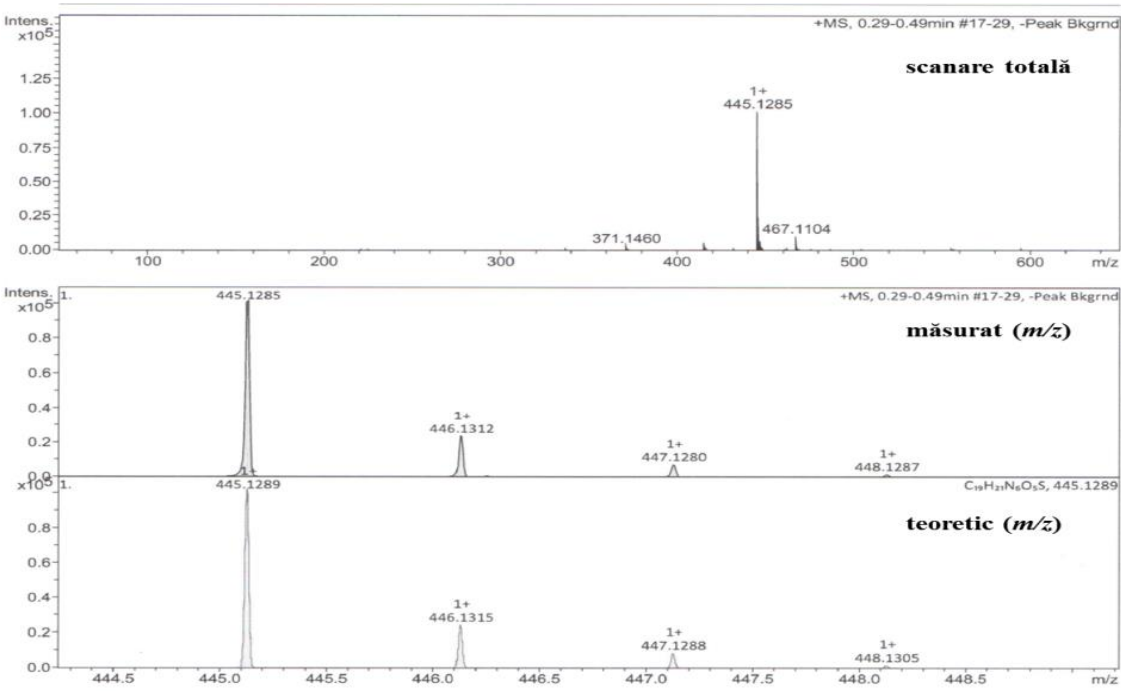

HRMS spectra of **6k**
